# Nonribosomal Peptides Produced by Minimal and Engineered Synthetases with Terminal Reductase Domains

**DOI:** 10.1002/cbic.202000176

**Published:** 2020-06-25

**Authors:** Andreas Tietze, Yan‐Ni Shi, Max Kronenwerth, Helge B. Bode

**Affiliations:** ^1^ Fachbereich Biowissenschaften, Molekulare Biotechnologie Goethe-Universität Frankfurt 60438 Frankfurt am Main Germany; ^2^ Buchmann Institute for Molecular Life Sciences (BMLS) Goethe-Universität Frankfurt 60438 Frankfurt am Main Germany; ^3^ Senckenberg Gesellschaft für Naturforschung 60325 Frankfurt Germany

**Keywords:** aldehydes, natural products, nonribosomal peptide synthetases, NRPS engineering, reductases

## Abstract

Nonribosomal peptide synthetases (NRPSs) use terminal reductase domains for 2‐electron reduction of the enzyme‐bound thioester releasing the generated peptides as C‐terminal aldehydes. Herein, we reveal the biosynthesis of a pyrazine that originates from an aldehyde‐generating minimal NRPS termed ATRed in entomopathogenic *Xenorhabdus indica*. Reductase domains were also investigated in terms of NRPS engineering and, although no general applicable approach was deduced, we show that they can indeed be used for the production of similar natural and unnatural pyrazinones.

## Graphical abstract

**Peptide aldehyde production**: An aldehyde‐releasing reductase (R) domain has been identified as part of a minimal nonribosomal peptide synthetase (NRPS) in *X. indica*. We also show that the R domain from the tilivalline‐producing NRPS can be used in engineered synthetases to introduce an aldehyde group and subsequently produce natural and unnatural pyrazinones.

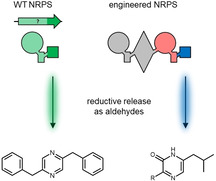

## Introduction

In the early 1960s, peptides were discovered that originate from a mechanism different from that of protein synthesis.^1^ These nonribosomal peptides (NRPs) show high structural diversity leading to many different biological activities exemplified by the clinically used antibiotic bacitracin, the anticancer agent bleomycin or the immunosuppressant cyclosporine.^2^ Their biosynthetic machinery can be found across all three domains of life,^3^ and today major insights into the underlying biochemistry and structural basis have been gained.^4^, ^5^

The assembly line‐fashioned biosynthesis of NRPs is carried out by large multifunctional nonribosomal peptide synthetases (NRPSs) which harbour a modular architecture.^2^, ^4^ Within one module, the adenylation (A) domain recognises and activates a specific amino acid (AA) under ATP consumption, which is then transferred to the 4’‐phosphopantetheinyl moiety of a post‐translationally modified peptidyl carrier protein also called thiolation (T) domain. The condensation (C) domain forms the peptide bond between two adjacent T domain‐bound AAs donating the nascent peptide chain to the following module, where it can be further elongated. Beside this multimodular system, also monomodular,^6^ NRPS‐like or minimal NRPSs lacking a C domain^7^ or even stand‐alone domains^8^ are known and commonly found in bacteria.^3^ Additionally, optional domains, for example, for fatty acid attachment, methylation, cyclization or epimerization of AAs and no restriction to the 20 proteinogenic AAs leads to aforementioned structural diversity.^2^ Instead of the most prevalent terminal thioesterase (TE) domains, which release the peptide chain from the NRPS, reductase (R) domains can be an alternative route for peptide release.^9^ They catalyse an NAD(P)H dependent two‐electron reduction of the thioester to an aldehyde which can be further reduced to an alcohol.^10^ Due to their electrophilic properties, aldehydes can contribute as intermediates, for example, for imine formation and subsequent modification as in tilivalline biosynthesis^11^ or are often associated with protease inhibitors, for example, by reversible binding of the active site's threonine of the *Mycobacterium tuberculosis* proteasome by fellutamide B.^12^

To get access to more NRPs that either can be modified to improve biological properties, circumvent bacterial resistances or are completely *de novo* peptides, engineering of NRPSs is a powerful tool.^13^ Since 1995,^14^ this has been the focus of many groups but no general applicable guidelines for NRPS engineering have been established.^15^ We recently introduced the concept of exchange units (XU), defining three rules for reproducible NRPS engineering: 1) the tridomain A−T−C is used as XU, 2) the C domain's acceptor site specificity has to be considered and 3) the conserved WNATE sequence depicts the fusion point within the flexible linker connecting the C and A domain.^16^ An improved technique (XUC) circumvents the limitation of the C domain specificity by using a fusion point within the linker connecting both subdomains of the C domain.^17^ Although the use of TE and even C domains have been investigated as termination domains, the final step within NRP biosynthesis remains a challenging factor in NRPS engineering. Furthermore, aldehyde‐generating R domains would provide an alternative route for peptide release and would increase structural diversity. Here, we describe the discovery of an R domain‐containing minimal NRPS and show examples of R domains in engineered NRPSs.

## Results and Discussion

**Figure 1:**
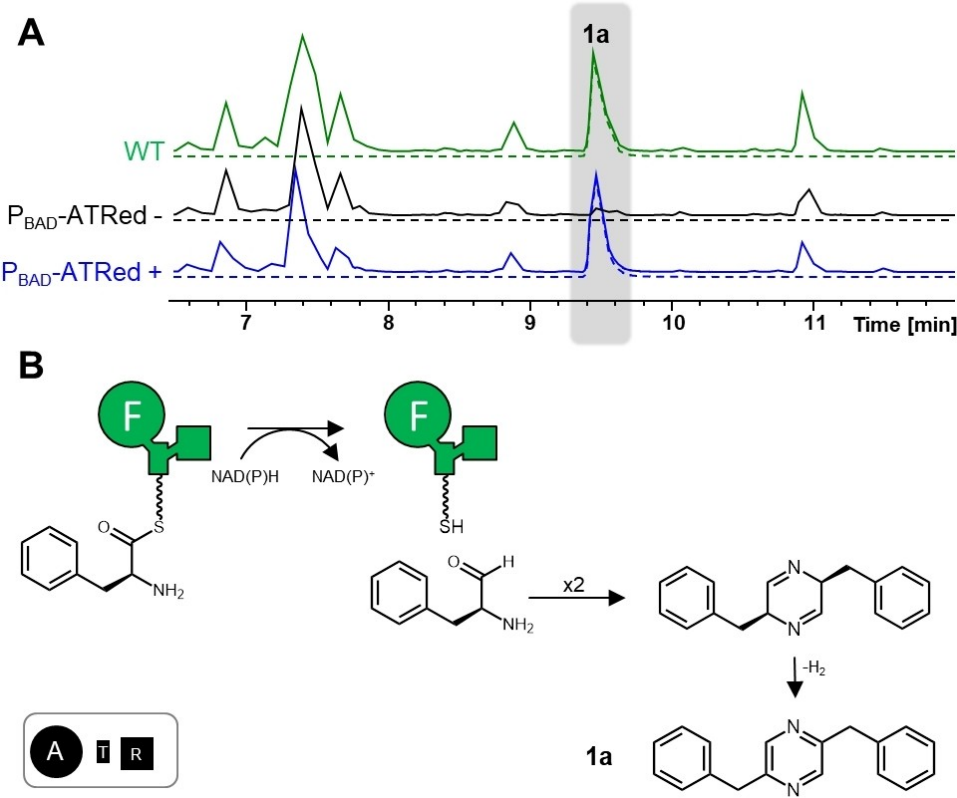
The ATRed NRPS in *X. indica*. A) High‐resolution LC–MS analysis of *X. indica* WT (green), uninduced promoter exchange mutant (black) and induced promoter exchange mutant (blue). The base peak chromatogram (BPC) is indicated by continuous lines, and the extracted ion chromatogram (EIC; **1 a**; *m/z* [*M*+H^+^]^+^ =261.13) by dashed lines. B) Proposed biosynthesis and structure of **1 a**. The ATRed consists of an A (large circle with activated AA substrate indicated by one‐letter code; here F), a T (rectangle) and an R (small square) domain.

AntiSMASH analysis^18^ identified a biosynthetic gene *xind01729* in the genome of the entomopathogenic *Xenorhabdus indica* DSM 17382 encoding a monomodular NRPS with a predicted terminal R domain that was not linked to any natural product (Figure S1 in the Supporting Information). Due to the domain arrangement of an A, T and R domain, this minimal NRPS was termed ATRed. Such a three domain architecture has already been described in, say, the biosynthesis of chloramphenicol in *Streptomyces*,^19^ virulence factors in *Pseudomonas*,^20^ piperazines in *Aspergillus* 21 and for CAR enzymes – a distant relative to the NRPS family – ‐responsible for the reduction of carboxylic acid substrates to the corresponding aldehydes in bacteria and fungi.^22^ An exchange of the promoter upstream of *xind01729* against an arabinose‐inducible promoter (P_BAD_) showed that compound **1 a** is associated with the encoded ATRed in the induced *X. indica* mutant compared to the uninduced mutant (Figures 1A and S2). The production of **1 a** was also observed upon heterologous expression of *xind01729* in *Escherichia coli* (Figure S3). Isolation and NMR analysis of **1 a** confirmed a structure of a pyrazine that is produced with a titre of 2.1±0.5 mg/L in the wild‐type strain (Figures S4–10, Table S4). Based on the domain arrangement and structure, we propose that phenylalanine is activated by the A domain, bound as thioester to the T domain and from there released as aldehyde by the R domain. Two amino aldehydes then form a cyclic Schiff base which subsequently oxidizes to a pyrazine (Figure 1B). This NRPS‐mediated pyrazine biosynthesis is also known from other R domain‐containing NRPSs.^20^, ^21^, ^23^ Furthermore, pyrazine derivatives with tryptophan (**1 b**) or tyrosine (**1 c**) instead of one phenylalanine residue were detected in small amounts suggesting a slightly relaxed A domain specificity (Figures S2 and S3) as well as a pyrazinone side product (**1 d**) made of two phenylalanines in *E. coli* (Figure S3).

**Figure 2:**
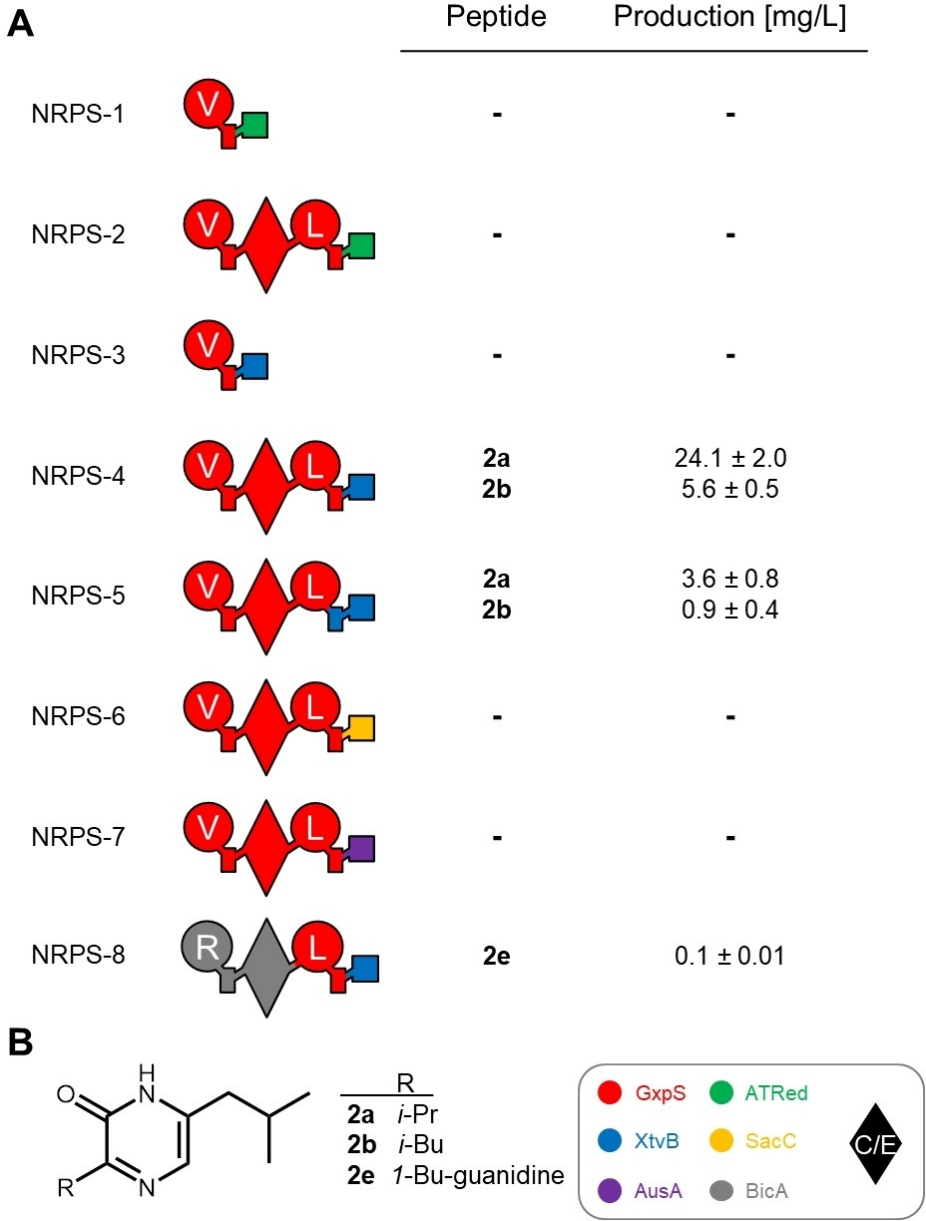
R domains for peptide release in engineered NRP biosynthesis. A) Schematic representation of engineered NRPSs with different R domains and peptide production as determined in triplicate. B) Structures of **2 a**, **2 b** and **2 e**. See Figure 1 for assignment of the domain symbols; further symbol: dual condensation/epimerization (C/E; diamond) domain. The colour code at the bottom identifies NRPSs used as building blocks (Figure S9).

Next, our aim was to analyse the potential application of R domains as release mechanism in engineered NRPS systems. Therefore the identified ATRed_*xind01729*__R domain was fused with the initiation module of the GameXPeptide‐producing NRPS (GxpS) in *Photorhabdus laumondii* subsp. *laumondii* TTO1^24^ (Figure S11) to keep the overall protein architecture (NRPS‐1, Figure 2a). This construct was also elongated by one GxpS module to a bimodular NRPS (NRPS‐2) similar to the NRPS involved in the biosynthesis of aureusimine in *Staphylococcus aureus*.^25^, ^26^ The engineered proteins were heterologously expressed in *E. coli* but, despite the presence of the expected proteins (Figure S12), no production of peptides was observed after LC–MS analysis (Figure 2A). We also tested the R domain from the tilivalline‐producing NRPS (XtvB) in *Xenorhabdus eapokensis* DL20^11^ instead of ATRed_*xind01729*__R with the initiation module as well as the first two modules from GxpS (NRPS‐3 and ‐4). In contrast to the monomodular NRPS‐3, bimodular NRPS‐4 produced compounds **2 a** and **2 b** with yields up to 24.1 mg/L (Figure S13). NMR analysis of the purified compound **2 a** (Figure S14‐20, Table S5) confirmed the structure of a 3‐isopropyl‐6‐isobutyl‐pryrazin‐2(1*H*)‐one (Figure 2B), and the appearance of two derivatives with valine and leucine as first amino acid is in line with the substrate promiscuity of the GxpS_A1 domain for both AAs.^24^ Due to the NRPS architecture and NRP structure, we assume an aureusimine‐like biosynthesis via a T‐domain‐bound dipeptide thioester that is reduced by XtvB_R, thus enabling intramolecular condensation of the generated aldehyde **2 c** with its amino group to a cyclic imine and subsequent oxidation to **2 a** and **2 b** (Figure S21).^25^ The aldehyde intermediate **2 c** was confirmed by using *O*‐(2,3,4,5,6‐pentafluorobenzyl)hydroxylamine (PFBHA;^27^ Figure S22).

Recent work showed that retaining the natural T−R interface in bacterial hybrid CAR enzymes leads to higher *k*_cat_ values.^28^ However, a version of NRPS‐4 maintaining the T−R didomain from XtvB (NRPS‐5) results in an approximately 6.5‐fold lower production of both derivatives. Preservation of the natural A−T didomain has also been reported previously in engineered NRPS systems with A−T−TE architecture.^29^ Furthermore, the fusion point C‐terminal to the last helix of the T domain used in this work (Figure S23) was shown in our development of the XU concept to be applicable for introducing terminal C domains for peptide release.^16^ Beyond ATRed_*xind01729*__R and XtvB_R, two more R domains from the postulated safracin‐producing NRPS (SacC)^30^ in *Xenorhabdus* sp. TS4 as well as the aureusimine‐producing NRPS (AusA) in *Staphylococcus lugdunensis* IVK28 (NRPS‐6 and −7) were tested with an analogous domain architecture to NRPS‐4 (Figure 2A). SacC processes 3‐hydroxy‐5‐methyl‐*O*‐methyltyrosine whereas AusA_R has been reported to accept a wide variety of substrates (Figure S11).^31^ Unfortunately, no production was observed. This suggests that domain‐domain interaction or the R domain's substrate specificity might be crucial for NRPS engineering with R domains as addressed in a molecular docking analysis of a T−R didomain^32^ and shown as a common limiting factor for engineering approaches.^15^

Due to the fact that XtvB_R does not exhibit strict substrate specificity (the domain reduces 3‐hydroxy anthranilic acid‐proline as part of the tilivalline biosynthesis and valine/leucine‐leucine in NRPS‐4) and the unnatural interaction with GxpS_T2 lead to good production titre, we modified NRPS‐4 at its N‐terminal position. Exchange of the first d‐valine‐/leucine‐specific XU against the d‐arginine specific XU from the bicornutin‐producing NRPS (BicA)^33^ in *Xenorhabdus budapestensis* DSM 16342 (NRPS‐8) resulted in the expected compound **2 e** (Figure S24). This was verified by labelling experiments (Figure S25) and comparison to a synthetic NMR standard (Figures S26–31, Table S6).

## Conclusion

Although NRPs with aldehydes are relatively rare, their appearance has often been reported with bioactivity like the cysteine protease and proteasome inhibitor flavopeptin from *Streptomyces*.^34^ In *Staphylococcus*, an R domain‐derived aldehyde serves as important intermediate in the biosynthesis of lugdunin, a promising novel antibiotic against methicillin‐resistant *S. aureus*.^35^ In this study, we identified the biosynthetic gene responsible for pyrazine biosynthesis in *X. indica* through an R domain containing minimal NRPS termed ATRed. The function of the NRP has not been addressed; however, compounds with pyrazine structures are shown to fulfil biological functions like cell‐to‐cell communication,^36^ thus qualifying them for further studies in order to elucidate their biological purpose. R domains were subsequently tested in engineered NRPSs and we could show that the R domain from the tilivalline‐producing NRPS can be used to introduce an aldehyde group in unnatural NRPs. Along with other NRPS engineering approaches, this allows the NRP to be further modified. Nevertheless, the majority of our engineered NRPSs were nonfunctional, thus suggesting that NRPS engineering with terminal R domains is not (yet) generally applicable and further experiments are needed. The limiting factor is probably due to substrate specificity or domain interactions; an issue which should be investigated more in detail with resolving the structure of a T−R didomain and further enzyme/cultivation optimisation.^23^, ^37^

## Experimental Section

**Strain cultivation**: All *E. coli* and *X. indica* strains (Table S1) were grown in liquid or solid lysogeny broth (LB; pH 7.5, 10 g/L tryptone, 5 g/L yeast extract and 5 g/L NaCl). Solid medium contained 1.5 % (*w*/*v*) agar. *Saccharomyces cerevisiae* strain CEN.PK 2‐1 C and derivatives were grown in liquid and solid yeast extract peptone dextrose (YPD) medium (10 g/L yeast extract, 20 g/L peptone and 20 g/L glucose). Agar plates contained 1.5 % (*w*/*v*) agar. Kanamycin (50 μg/mL) and G418 (200 μg/mL) were used as selection markers. *E. coli* was cultivated at 37 °C, and all other strains were cultivated at 30 °C. *E. coli* ST18 cells were supplemented with 50 μg/mL 5‐aminolevulinic acid. For production of **1 a**, *X. indica* was inoculated from an overnight culture in 10 mL volume and grown for 48 h at 160 rpm with 2 % (*v*/*v*) Amberlite XAD‐16. P_BAD_ promoters were induced with 0.02 % l‐arabinose. For the detection of aldehydes,^27^ 0.2 mM PFBHA was added to the LB culture.

**Generation of promoter exchange mutants**: The first 700 bp of *xind01729* were cloned in the PCR‐amplified backbone of pCEP_Kan^38^ and *E. coli* ST18 cells were transformed with the plasmid. ST18 and *X. indica* wildtype cells were grown in 10 mL LB from an overnight culture to an OD_600_ of 0.6–0.8, washed twice and mixed on an LB plate without 5‐aminolevulinic acid in a ratio of 1 : 3. After incubation for 24 h at 30 °C, the cells were harvested and incubated for another 72 h on selection medium containing kanamycin.

**Cloning of plasmids and transformation of cells**: Genomic DNA of *Xenorhabdus* and *Photorhabdus* strains was isolated using the Qiagen Gentra Puregene Yeast/Bact Kit. Genomic DNA of *S. lugdunensis IVK28* was provided by B. Krismer (Eberhard Karls University of Tübingen, Germany). PCR was performed with oligonucleotides obtained from Eurofins Genomics (Table S3). Cloning was done by Hot fusion^39^ or transformation‐associated recombination (TAR),^40^ and the fragments were amplified in a two‐step PCR program with homology arms (20 or 40–80 bp, respectively). For PCR, S7 Fusion high‐fidelity DNA polymerase (Biozym) and Q5 high‐fidelity DNA polymerase (New England BioLabs) were used according to the manufacturers’ instructions. The vector pFF1 was digested with EcoRI and SgsI. All fragments were digested with DpnI (Thermo Fisher Scientific). DNA purification was performed with MSB® Spin PCRapace (STRATEC Biomedical AG) or from 1 % TAE agarose gel using Invisorb® Spin DNA Extraction (STRATEC Biomedical AG). Plasmids (Table S2) were transformed into *E. coli* DH10B::mtaA by electroporation and verified by restriction digest. Plasmid was isolated from *E. coli* by using Invisorb® Spin Plasmid Mini Two (STRATEC Biomedical AG).

**Heterologous expression of NRPSs and extract preparation**: *E. coli* cells harbouring the constructed plasmids were inoculated from an overnight culture to 10 mL cultures containing 2 % (*v*/*v*) Amberlite XAD‐16, kanamycin and arabinose for 48 h at 22 °C and 160 rpm.

The XAD‐16 beads were harvested by sieving and incubated with one culture volume MeOH for 30 min at 160 rpm. The organic phase was filtered and evaporated to dryness under reduced pressure as described before.^17^ Extracts were solved in 1 mL MeOH and diluted 1 : 10 for LC‐MS measurements.

**LC–MS analysis**: All measurements were carried out by using an Ultimate 3000 LC system (Dionex; gradient of MeCN/0.1 % formic acid in H_2_O/0.1 % formic acid, 5 % to 95 %, 15 min, flow rate 0.4 mL/min, ACQUITY UPLC BEH C18 column 1.7 μm 2.1 mm×100 mm (Waters)) coupled to an AmaZonX (Bruker) electron spray ionization (ESI) mass spectrometer in positive ionization mode or to an Impact II qTof (Bruker) with internal 10 mM sodium formate calibrant for high‐resolution data. The software DataAnalysis 4.3 (Bruker) was used to evaluate the measurements.

**SDS‐PAGE analysis**: A 20 mL LB culture was inoculated to an OD_600_=0.05 with an overnight culture of *E. coli* cells with the respective NRPS‐expressing plasmid and was grown for 18 h at 160 rpm. Cells with IPTG‐inducible plasmids were grown at 37 °C until an OD_600_=0.7 for induction and subsequently grown at 16 °C; cells with arabinose‐inducible plasmids were grown at 26 °C and induced upon inoculation. The OD_600_ was normalized with LB, the cell pellet (3200 rpm, 10 min, 4 °C) of 20 mL was resuspended in 10 mL lysis buffer (100 mM HEPES pH 7.6, 200 mM NaCl, 0.1 % Triton X‐100, 1 mM dithiothreitol, 1 mM EDTA, protease inhibitor and lysozyme) and incubated for 20 min on ice. After sonication on ice, the supernatant (13 300 rpm, 15 min) was mixed with 3x loading buffer (100 mM Tris**⋅**Cl pH 6.8, 4 % (*w*/*v*) SDS, 0.2 % Bromophenol Blue, 200 mM β‐mercaptoethanol), incubated at 37 °C for 20 min and separated on 8 % SDS‐PAGE gels.

**Labeling experiments**: *E. coli* cells with the respective NRPS‐expressing plasmid were grown in ISOGRO®‐^13^C or ‐^15^N (Sigma–Aldrich) medium.^24^ 2 mM unlabelled AA was added to the culture; cultivation and extract preparation were performed as described above.

**Peptide synthesis, purification and quantification**: Compound **2 e** was chemically synthesized as described by Schilling et al. by using H‐Leu‐H NovaSyn TG resin (15.8 μmol, Sigma‐Aldrich) and Fmoc‐D‐Arg(Pbf)‐OH (63 μmol, Iris Biotech) with 1‐[bis(dimethylamino)methylene]‐1*H*‐1,2,3‐triazolo[4,5‐*b*]pyridinium 3‐oxid hexafluorophosphate (HATU; 63 μmol, Carbolution), 1‐hydroxy‐1*H*‐benzotriazole (HOBt; catalytic, Sigma‐Aldrich) and NMM (126 μmol, Sigma–Aldrich) in ACN for 1 h coupling reaction.^41^ After Fmoc deprotection with 20 %(*v*/*v*) piperidin (Iris Biotech) in DMF, cyclization occurred after cleavage from the resin (79.95 % ACN/20 % water/0.05 % TFA (*v*/*v*/*v*)) and the Pbf group was finally deprotected with TFA.

Compounds **1 a**, and **2 a** were purified from 1 L culture by using a 1260 Infinity II LC system and 1260 Semiprep LC system (Eclipse XDB−C18 7 μm 21.2×250 mm) coupled to a G6125B LC/MSD ESI‐MS (Agilent). Synthesised **2 e** was purified by using a 1260 Infinity II LC system (Agilent).

All peptides were quantified in triplicates using a calibration curve (11 values ranging from 100 μg/mL to 0.02 μg/mL) and HPLC‐MS measurements. As standards, purified **1 a** (for quantification of **1 a**), **2 a** (for quantification of **2 a** and **2 b**) and synthetic **2 e** (for quantification of **2 e**) were used.

**NMR analysis**: Structures of **1 a**, **2 a** and **2 e** were elucidated by 1D and 2D NMR experiments. ^1^H, ^13^C, COSY, HSQC and HMBC spectra were measured on a Bruker AV500 spectrometer using CD_3_OD and [D_6_]DMSO as solvent.^17^ Coupling constants are expressed in Hz and chemical shifts are given on a ppm scale.

## Conflict of interest

The authors declare no conflict of interest.

## Supporting information

As a service to our authors and readers, this journal provides supporting information supplied by the authors. Such materials are peer reviewed and may be re‐organized for online delivery, but are not copy‐edited or typeset. Technical support issues arising from supporting information (other than missing files) should be addressed to the authors.

Supplementary
