# Supplementary material for: Nonribosomal Peptides Produced by Minimal and Engineered Synthetases with Terminal Reductase Domains
Source: Chembiochem. 2020 Jun 25;21(19):2750–4. doi: 10.1002/cbic.202000176 (PMC7586950; doi:10.1002/cbic.202000176)
Supplement: Supplementary file 1 — Supplementary [file CBIC-21-2750-s001.pdf]

ChemBioChem

Supporting Information

Nonribosomal Peptides Produced by Minimal and Engineered Synthetases with Terminal Reductase Domains

Andreas Tietze, Yan-Ni Shi, Max Kronenwerth, and Helge B. Bode*

Supplementary Table 1. Strains used and generated in this work.

Strain	Genotype	Reference
E. coli BL21 DE3	F– ompT hsdSB(rB- mB-) gal dcm lon λ(DE3 [lacI lacUV5-T7 gene 1 ind1 sam7 nin5])	Invitrogen
E. coli BL21 DE3 pET11a_*xind01729* pCK_mtaA	*E. coli* BL21star DE3 pET11a_*xind01729* pCK_mtaA, Amp^R, Cm^R	This work
E. coli DH10B	F_ mcrA (*mrr*-*hsd*RMS-*mcr*BC), 80*lac*ZΔ, M15, Δ*lac*X74 *rec*A1 *end*A1 *ara*D 139 Δ(*ara, leu*)7697 *gal*U *gal*K λ*rpsL* (*Strr*) *nup*G	[1]
E. coli DH10B::mtaA	DH10B with mtaA from pCK_mtaA ΔentD	[2]
E. coli ST18		[3]
E. coli ST18 pCEP-Kan_*xind01729*	*E. coli* ST18 pCEP-Kan_*xind01729,* Kan^R	This work
S. cerevisiae CEN.PK 2-1C	MATa; his3D1; leu2-3_112; ura3-52; trp1-289; MAL2-8c; SUC2	Euroscarf
P. luminescens TT01		DSMZ
Xenorhabdus sp. TS4		DSMZ
X. eapokensis DL20		DSMZ
X. budapestensis DSM 16342		DSMZ
X. indica DSM 17382		DSMZ
X. indica DSM 17382::pCEP-Kan_*xind01729*	*X. indica* DSM 17904::pCEP-Kan_*xind01729,* Kan^R	This work
E. coli DH10B::mtaA pAT41_*NRPS-1*	*E. coli* DH10B::mtaA pAT41_*NRPS-1*, Kan^R	This work
E. coli DH10B::mtaA pAT41_*NRPS-2*	*E. coli* DH10B::mtaA pAT41_*NRPS-2*, Kan^R	This work
E. coli DH10B::mtaA pAT41_*NRPS-3*	*E. coli* DH10B::mtaA pAT41_*NRPS-3*, Kan^R	This work
E. coli DH10B::mtaA pAT41_*NRPS-4*	*E. coli* DH10B::mtaA pAT41_*NRPS-4*, Kan^R	This work
E. coli DH10B::mtaA pAT41_*NRPS-5*	*E. coli* DH10B::mtaA pAT41_*NRPS-5*, Kan^R	This work
E. coli DH10B::mtaA pAT41_*NRPS-6*	*E. coli* DH10B::mtaA pAT41_*NRPS-6*, Kan^R	This work
E. coli DH10B::mtaA pAT41_*NRPS-7*	*E. coli* DH10B::mtaA pAT41_*NRPS-7*, Kan^R	This work
E. coli DH10B::mtaA pAT41_*NRPS-8*	*E. coli* DH10B::mtaA pAT41_*NRPS-8*, Kan^R	This work

Supplementary Table 2. Oligonucleotides used in this work.

Plasmid	Oligonucleotide	Sequence (5'->3')	Template
pCEPKan_*xind01729*	pCEP_fw_gib	ATGTGCATGCTCGAGCTC	pCEP-Kan
	pCEP_rv_gib	ATGCTAGCCTCCTGTTAGC	
	PF_7	TTTTGGGCTAACAGGAGGCTAGCATATGATAAATACCACCCCCTATAATTTCG	X. indica DSM 17382
	PF_8	ATCTGCAGAGCTCGAGCATGCACATCGTGGCCGTCATAATCAGAC	
pET11a_*xind01729*	pET11a_for_strep-tag	GTAGGATGGAGCCATCCACAGTTCGAGAAGTAAGGATCCGGCTGCTAAC	pET11a-modified
	pET11a rev	ATGTATATCTCCTTCTTAAAGTTAAACAAAATTATTTCTA	
	PF_15	TTAACTTTAAGAAGGAGATATACATATGATAAATACCACCCCCTATAATTTCG	*X. indica* DSM 17382
	PF_16	CGAACTGTGGATGGCTCCATCCTACTGATAAAAAACCTATTTTTTTCCAGTAAGTAAGATAAC	
pAT41_*NRPS-1*	AT_293	GATACCTATCTGAATAGTGATAAAAAATCAAATAATG	pAT41_*NRPS-2*
	AT_470	TCAGATTTCGTGATGTTCGTC	
	AT_471	ACGAACATCACGAAATCTGACGCTCAAATCAGTGGTGGC	pAT41_*NRPS-4*
	AT_483	TCACTATTCAGATAGGTATCCAATGTTTGGGCCAACTCCG	
pAT41_*NRPS-2*	pAT41_bb+Ypet_fw	GGATCCGCTGGCTCC	pAT41_*NRPS-4*
	AT_451	GATTTTCTCGGTAAATGTCGCC	
	AT_454	ATTGGCGACATTTACCGAGAAAATCCAACAAAACAAGAACGAGCTCACTG	*X. indica* DSM 17382
	AT_455	AACCAGCAGCGGAGCCAGCGGATCCCTATGATAAAAAACCTATTTTTTTCCAGTAAGTAAGATAAC	
pAT41_*NRPS-3*	AT_293	GATACCTATCTGAATAGTGATAAAAAATCAAATAATG	pAT41_*NRPS-4*
	AT_470	TCAGATTTCGTGATGTTCGTC	
	AT_471	ACGAACATCACGAAATCTGACGCTCAAATCAGTGGTGGC	pAT41_*NRPS-4*
	AT_483	CGGTGATGTTCTGTCTGGTCTACACTCAGAGTCTGGGCGACAAA	
pAT41_*NRPS-4*	pAT41_bb+YPet_fw	GGATCCGCTGGCTCC	*pAT41*
	pAT41_bb_rv	GGAATTCCTCCTGTTAGCCC	
	AL_GxpS-2-1	ACTGTTTCTCCATACCCGTTTTTTTGGGCTAACAGGAGGAATTCCATGAAAGATAGCATGGCTAAAAAGG	*P. luminescens* TT01
	AT_328	TTTCATTATTTGATTTTTTATCACTATTCAGATAGGTATCGATTTTCTCGGTAAATGTCGCC	
	AT_308	GATACCTATCTGAATAGTGATAAAAAATCAAATAATGAAATAAAAAAATAC	*X. eapokensis* DL20
	AT_289	TCATGAACTCGCCAGAACCAGCAGCGGAGCCAGCGGATCCCTTACTTTCAGGTTTATATGACGGTATGCTTG	
pAT41_*NRPS-5*	pAT41_bb+Ypet_fw	GGATCCGCTGGCTCC	pAT41_*NRPS-4*
	AT_458	CGCGACATAAATTTGGCGAG	
	AT_460	TTTTGCTCGCCAAATTTATGTCGCGGTTCTGACTTCAACCGAACAAACAG	*X. eapokensis* DL20
	AT_459	(AACCAGCAGCGGAGCCAGCGGATCCTTACTTACTTTCAGGTTTATATGACGGTATGC	
pAT41_*NRPS-6*	pAT41_bb+Ypet_fw	GGATCCGCTGGCTCC	pAT41_*NRPS-4*
	AT_451	GATTTTCTCGGTAAATGTCGCC	
	AT_452	ATTGGCGACATTTACCGAGAAAATCGAAATTTATCGGCGCGAAGG	*X. TS4*
	AT_453	AACCAGCAGCGGAGCCAGCGGATCCTTATTTCTGTTCCTGTGTCTGGTGTTG	
pAT41_*NRPS-7*	pAT41_bb+Ypet_fw	GGATCCGCTGGCTCC	pAT41_*NRPS-4*
	AT_451	GATTTTCTCGGTAAATGTCGCC	
	AT_456	ATTGGCGACATTTACCGAGAAAATCGAACAGCAAAGTGACGAATCGTG	*S. lugdunensis*
	AT_457	AACCAGCAGCGGAGCCAGCGGATCCTCATCTATGGTATTCTTTACATTCAAATTTTTCATTG	
pAT41_*NRPS-8*	pAT41_bb+YPet_fw	GGATCCGCTGGCTCC	pAT41_*NRPS-4*

pAT41_bb_rv	GGAATTCCTCCTGTTAGCCC	
JK-P1	CGGATCCTACCTGACGCTTTTTATCGCAACTCTCTACTGTTTCTCCATACCCGTTTTTTTGGGCTAACAGGAGG AATTCCATGAAAGATAACATTGCTACAGTGG	pFF1_*NRPS_5**
AT_328	TTTCATTATTTGATTTTTTTATCACTATTCAGATAGGTATCGATTTTCTCGGTAAATGTCGCC	
AT_308	GATACCTATCTGAATAGTGATAAAAAAATCAAATAATGAAATAAAAAAATAC	*X. eapokensis* DL20
AT_289	TCATGAACTCGCCAGAACCAGCAGCGGAGCCAGCGGATCCCTTACTTTCAGGTTTATATGACGGTATGCTTG	

Supplementary Table 3. Plasmids used and generated in this work. pFF1_*NRPS_5* from ([4]) is indicated with an * to avoid confusion with NRPSs constructed in this work.

Plasmid	Genotype	Reference
pAT41	2µ ori, URA3, P_{BAD} promoter, pCOLA ori, Ypet-Flag, Kan^R, MCS	[4]
pCK_mtaA	Cm^R, ori p15A, *mtaA*	[5]
pET11a-modified	pBR322 ori, P_{T7} promoter, Amp^R, lacI, His_6-smt3 tag	[6]
pET11a_*xind01729*	pBR322 ori, P_{T7} promoter, Amp^R, lacI, *xind01729*, strep tag	This work
pFF1	2µ ori, kanMX4, P_{BAD} promoter, pCOLA ori, Ypet-Flag, Kan^R, MCS	[7]
pFF1_*NRPS_5**	2µ ori, kanMX4, P_{BAD} promoter, pCOLA ori, Ypet-Flag, Kan^R, *bicA*-A1T1C2_*gxpS*-A2T2C3A3T3C4A4T4C_{Dsub}5_*bicA*-C_{Asub}5A5T5C_{term}	[4]
pCEP-Kan	R6Kγ ori, oriT, Kan^R, araC, P_{BAD} promoter	[8]
pCEP-Kan_*xind01729*	R6Kγ ori, oriT, Kan^R, araC, P_{BAD} promoter, *xind01729* (bp 1-700)	This work
pAT41_*NRPS-1*	2µ ori, URA3, P_{BAD} promoter, pCOLA ori, Ypet-Flag, Kan^R, *gxpS*_A1T2-*xind01729*_R	This work
pAT41_*NRPS-2*	2µ ori, URA3, P_{BAD} promoter, pCOLA ori, Ypet-Flag, Kan^R, *gxpS*_A1T2CE2A2T2-*xind01729*_R	This work
pAT41_*NRPS-3*	2µ ori, URA3, P_{BAD} promoter, pCOLA ori, Ypet-Flag, Kan^R, *gxpS*_A1T2-*xtvB*_R	This work
pAT41_*NRPS-4*	2µ ori, URA3, P_{BAD} promoter, pCOLA ori, Ypet-Flag, Kan^R, *gxpS*_A1T2CE2A2T2-*xtvB*_R	This work
pAT41_*NRPS-5*	2µ ori, URA3, P_{BAD} promoter, pCOLA ori, Ypet-Flag, Kan^R, *gxpS*_A1T2CE2A2-*xtvB*_T2R	This work
pAT41_*NRPS-6*	2µ ori, URA3, P_{BAD} promoter, pCOLA ori, Ypet-Flag, Kan^R, *gxpS*_A1T2CE2A2T2-*sacC*_R	This work
pAT41_*NRPS-7*	2µ ori, URA3, P_{BAD} promoter, pCOLA ori, Ypet-Flag, Kan^R, *gxpS*_A1T2CE2A2T2-*ausA*_R	This work
pAT41_*NRPS-8*	2µ ori, URA3, P_{BAD} promoter, pCOLA ori, Ypet-Flag, Kan^R, *bicA*_A1T2CE2-*gxpS*_A2T2-*xtvB*_R	This work

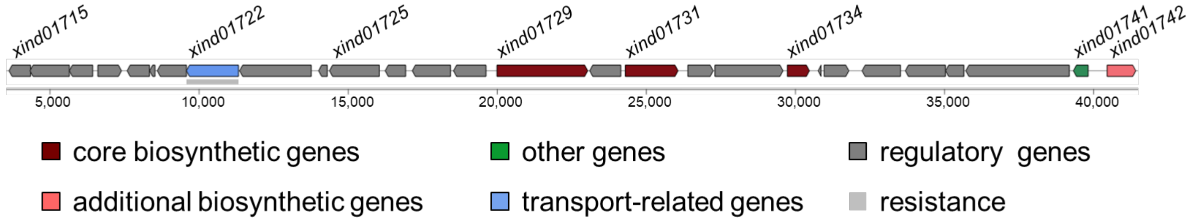

xind01715	condensin subunit E	***xind01729***	**ATRed**
xind01716	condensin subunit F	*xind01730*	L-asparaginase
xind01717	methyltransferase	*xind01731*	ribosomal protein S12 methylthiotransferase accessory factor
xind01718	membrane protein	*xind01732*	formate transporter
xind01719	3-deoxy-D-manno-octulosonate cytidylyltransferase	*xind01733*	formate acetyltransferase 3
xind01720	hypothetical protein	*xind01734*	pyruvate formate-lyase 1-activating enzyme
xind01721	tetraacyldisaccharide 4'-kinase	*xind01735*	hypothetical protein
xind01722	lipid A ABC transporter ATP-binding protein/permease	*xind01736*	membrane protein
xind01723	ComEC family protein	*xind01737*	serine-tRNA ligase
xind01724	DNA-binding protein HU	*xind01738*	Holliday junction DNA helicase
xind01725	30S ribosomal protein S1	*xind01739*	outer membrane lipoprotein carrier protein
xind01726	cytidylate kinase	*xind01740*	cell division protein FtsK
xind01727	3-phosphoshikimate 1-carboxyvinyltransferase	*xind01741*	AsnC family transcriptional regulator
xind01728	phosphoserine aminotransferase	*xind01742*	thioredoxin reductase

Supplementary Figure 1. Genomic region of *X. indica* DSM 17382 containing the ATRed encoding gene *xind01729*. The gene annotations and details are according to antiSMASH 5.1.2.[9]

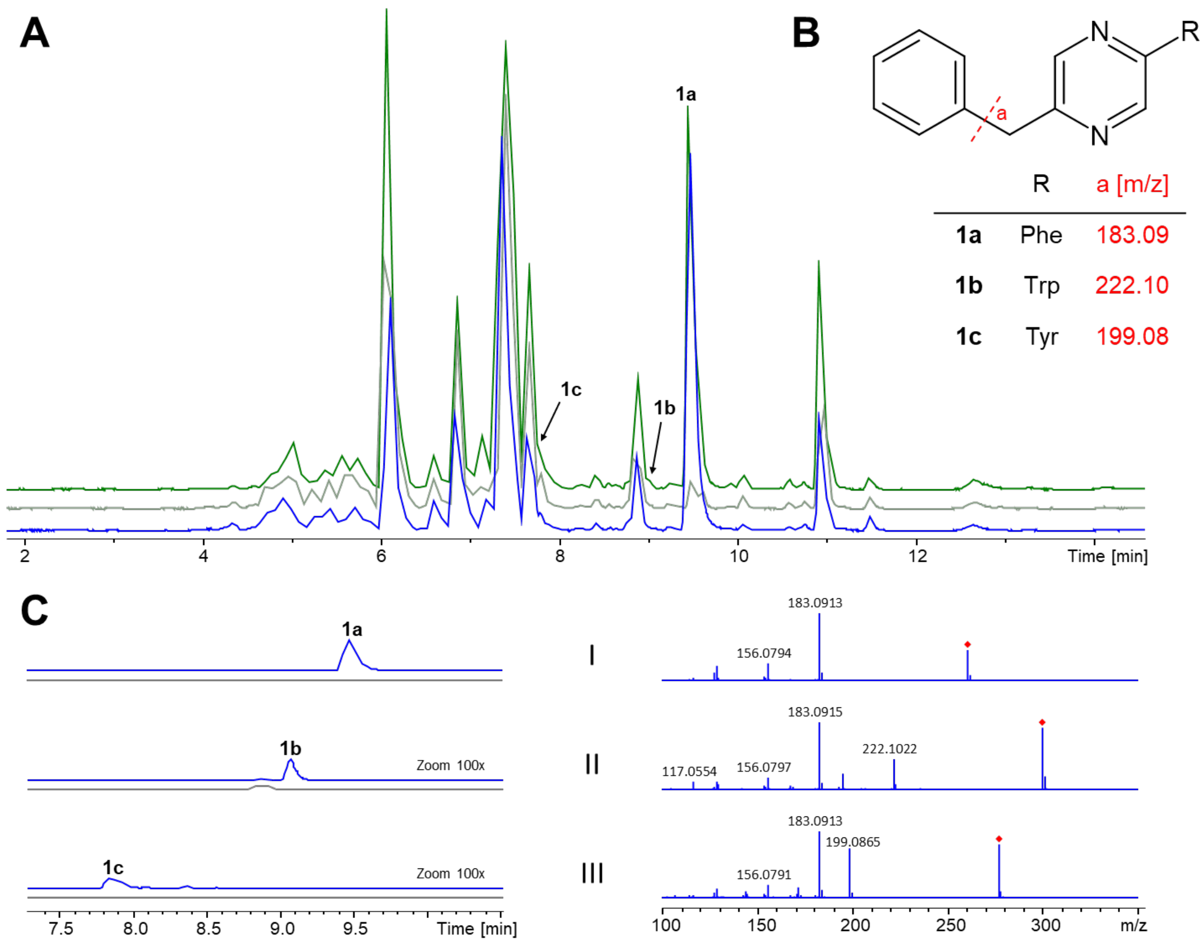

Supplementary Figure 2. HR-HPLC-MS data of **1a**, **1b** and **1c** produced by *X. indica* WT and promoter exchange mutant of *xind01729*. **(A)** Stacked BPC of production from *X. indica* WT (green) and promoter exchange mutant of *xind01729* (grey, non-induced; blue, induced). **(B)** Structure of **1a**, **1b** and **1c** and MS^2 fragments (red). **(C)** Stacked EIC (left) and MS^2 spectra (right) of **1a** (I, rt = 9.5 min, *m/z* $[M+H^+]^+$ = 261.138; calculated ion formula $C_{18}H_{17}N_2$; Δppm 1.4), **1b** (II, rt = 9.1 min, *m/z* $[M+H^+]^+$ = 300.149; calculated ion formula $C_{20}H_{18}N_3$; Δppm -0.3) and **1c** (III, rt = 7.9 min, *m/z* $[M+H^+]^+$ = 277.130; calculated ion formula $C_{18}H_{17}N_2O$; Δppm 0.5)

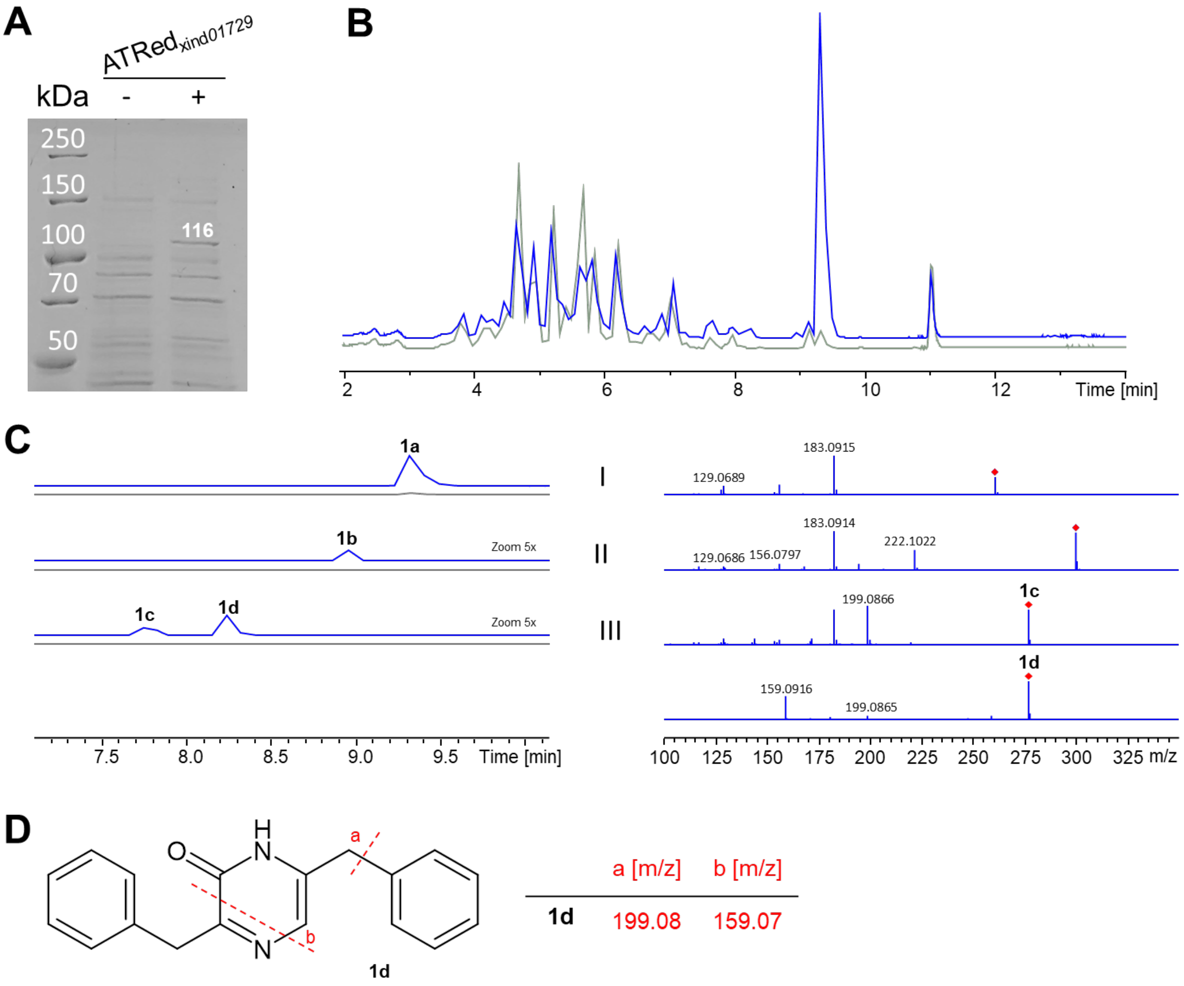

Supplementary Figure 3. HR-HPLC-MS data of **1a**, **1b**, **1c** and **1d** produced by ATRed*xind01729* after heterologous expression in *E. coli*. **(A)** SDS-PAGE analysis of protein extracts of non-induced (-) and induced (+) sample. The calculated molecular weights of the protein and the size of the marker proteins are indicated. **(B)** Stacked BPC of non-induced (grey) and induced (blue) production from ATRed*xind01729*. **(C)** Stacked EIC (left) and MS^2 spectra (right) of **1a** (I, rt = 9.4 min, *m/z* $[M+H^+]^+$ = 261.138; calculated ion formula $C_{18}H_{17}N_2$; Δppm 1.0), **1b** (II, rt = 9.0 min, *m/z* $[M+H^+]^+$ = 300.149; calculated ion formula $C_{20}H_{18}N_3$; Δppm 1.3), **1c** (III, rt = 7.8 min, *m/z* $[M+H^+]^+$ = 277.133; calculated ion formula $C_{18}H_{17}N_2O$; Δppm 0.0) and **1d** (III, rt = 8.3 min, *m/z* $[M+H^+]^+$ = 277.133; calculated ion formula $C_{18}H_{17}N_2O$; Δppm 0.8). **(D)** Postulated structure of **1d** and MS^2 fragments (red).

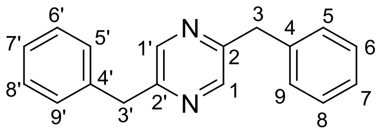

1a

Supplementary Figure 4. Structure of compound **1a**.

Supplementary Table 4. ^{1}H (500 MHz) and ^{13}C (125 MHz) NMR spectroscopic data for compound **1a** in DMSO-d_6 (δ in ppm and J in Hz).

no.	**1a**	
	δ_C	δ_H (mult., J)
1, 1'	143.7	8.30 (s)
2, 2'	153.7	
3, 3'	41.6	4.05 (s)
4, 4'	138.4	
5, 5', 9, 9'	129.0	7.18 (m)
6, 6', 8, 8'	128.8	7.22 (m)
7, 7'	126.7	7.15 (m)

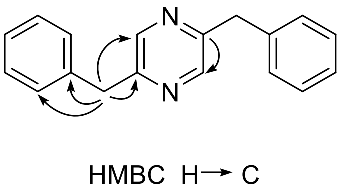

Supplementary Figure 5. Key HMBC correlations of **1**a.

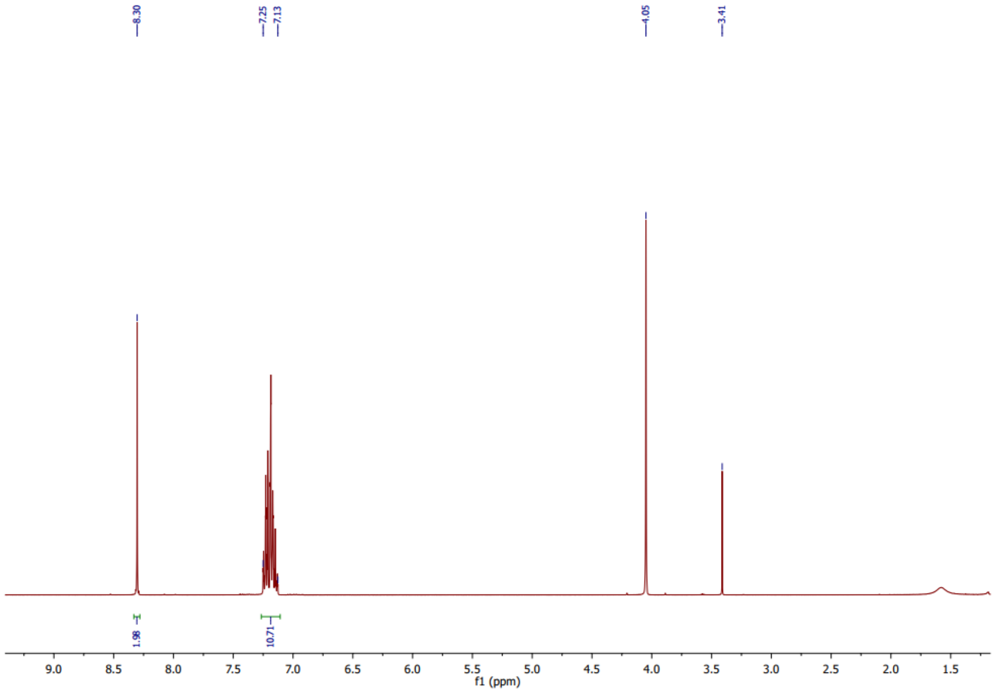

Supplementary Figure 6. ^{1}H NMR spectrum of compound **1a**.

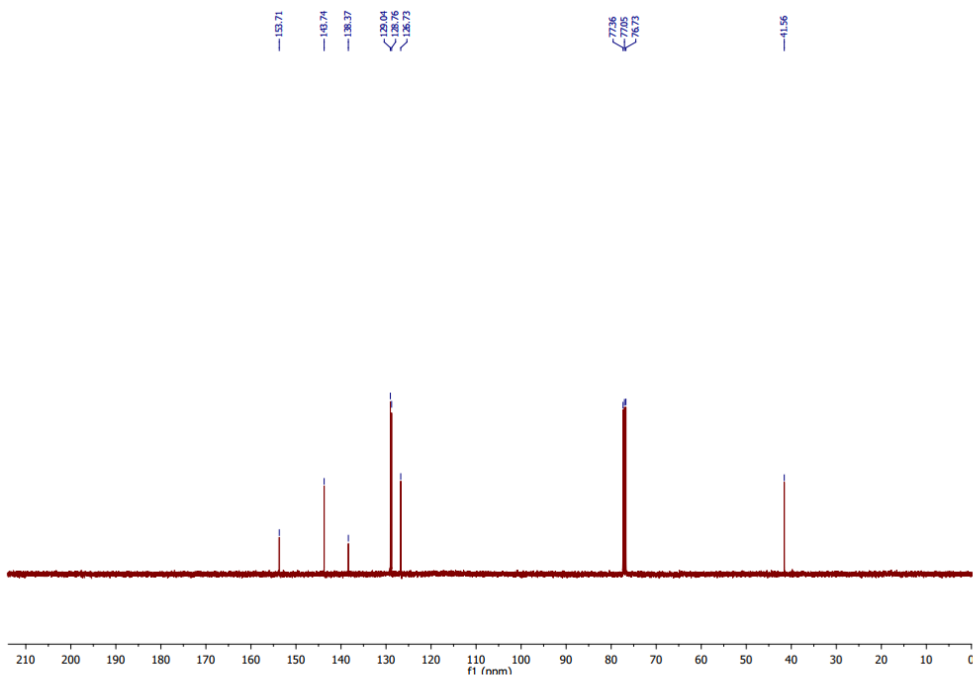

Supplementary Figure 7. ^{13}C NMR spectrum of compound **1a**.

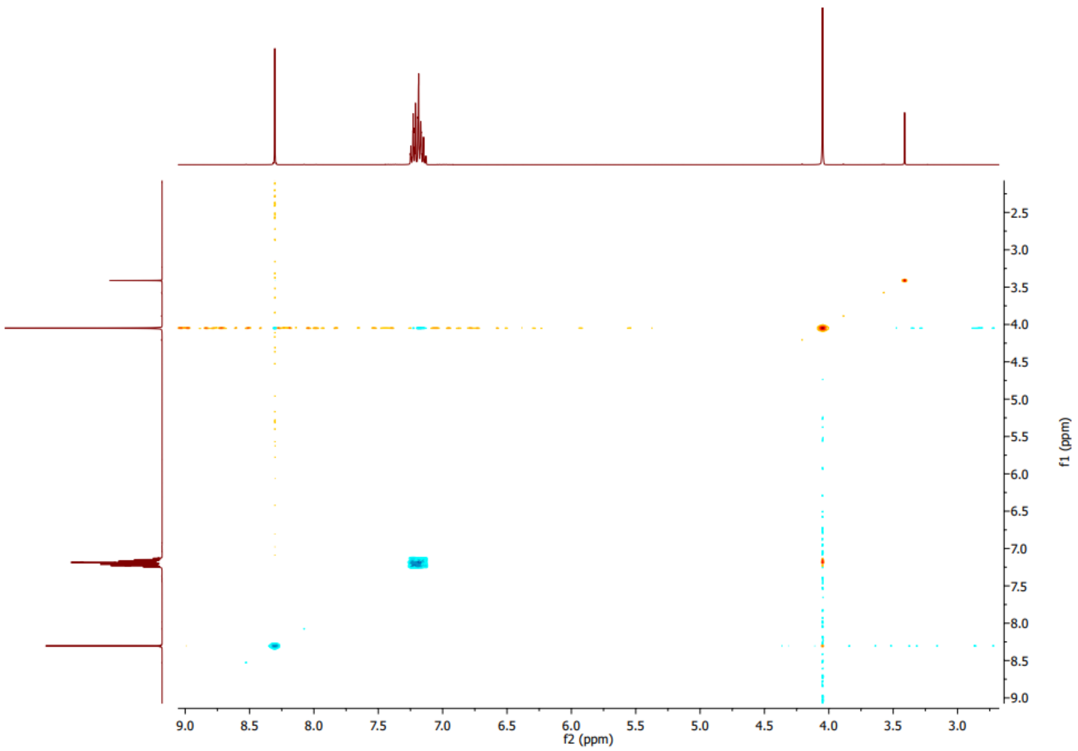

Supplementary Figure 8. COSY spectrum of compound **1a**.

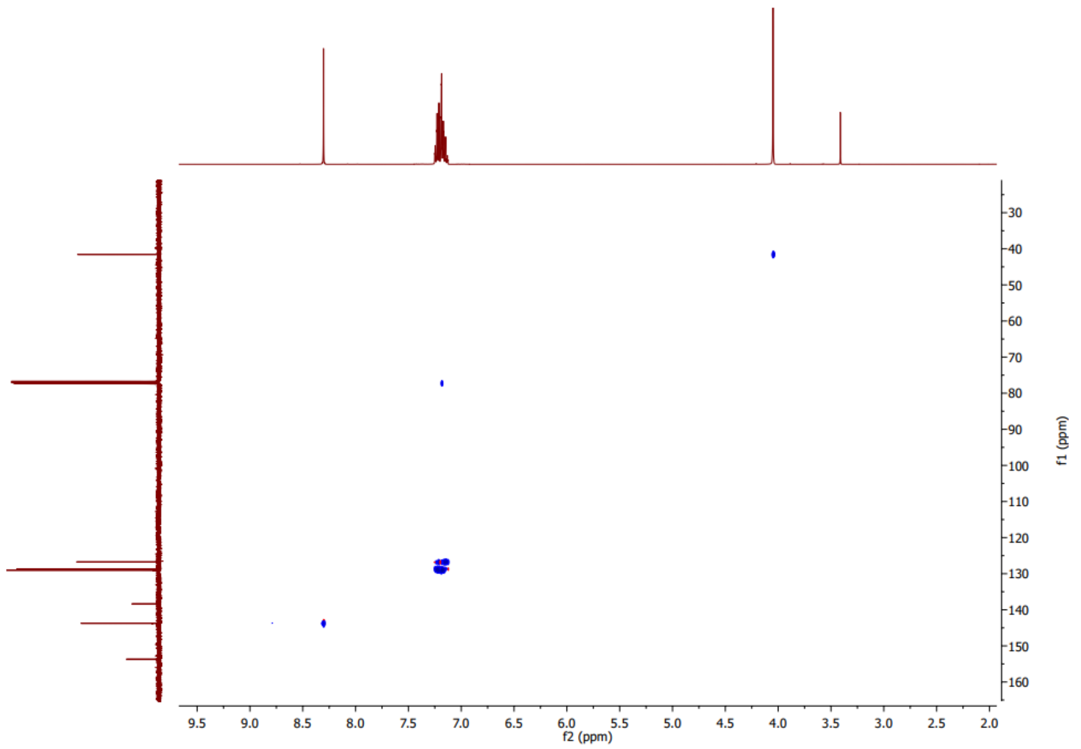

Supplementary Figure 9. HSQC spectrum of compound **1a**.

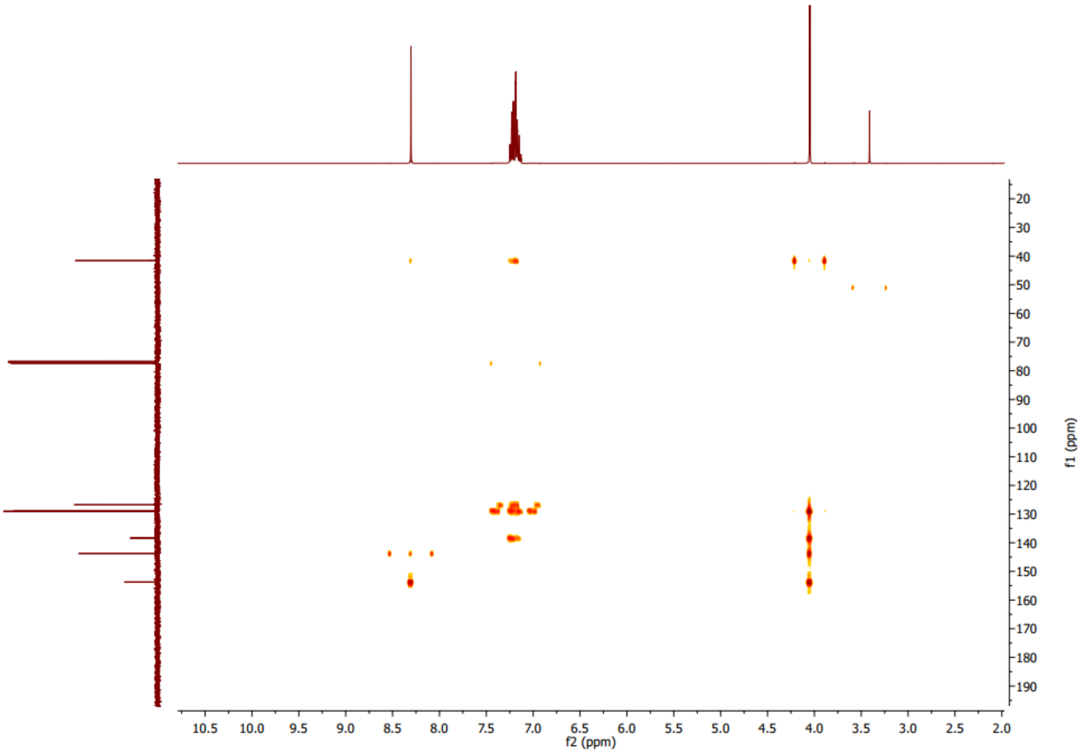

Supplementary Figure 10. HMBC spectrum of compound **1a**.

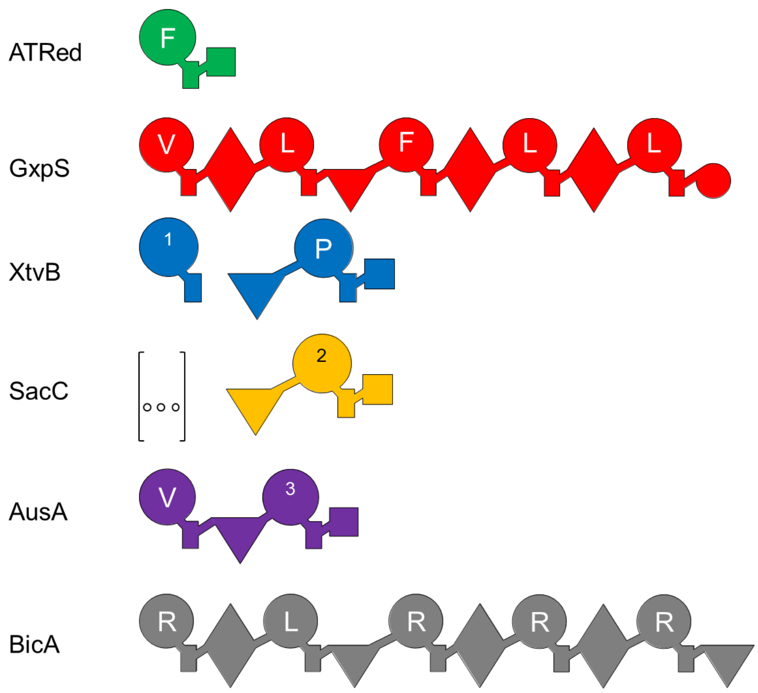

Supplementary Figure 11. Schematic overview of all NRPSs used in this work. ATRed$_{xind01729}$ from *X. indica* (WP_047678938), GxpS from *P. laumondii* subsp. *laumondii* TT01[10], XtvB from *X. eapokensis* DL20[11], SacC from *Xenorhabdus* sp. TS4 (PRJNA328577), AusA from *S. lugdunensis* (WP_012990658) and BicA from *X. budapestensis*[12]. Substrate specificities are assigned for all A domains with (1) as 3-hydroxy anthranilic acid, (2) as 3-hydroxy-5-methyl-*O*-methyltyrosine (3) as leucine, tyrosine, phenylalanine, 4-fluoro-phenylalanine, 4-chloro-phenylalanine, 3-chloro-tyrosine and (S)-(+)-a-amino-cyclohexane propionic acid. See Fig. 1 and 2 for assignment of the domain symbols.

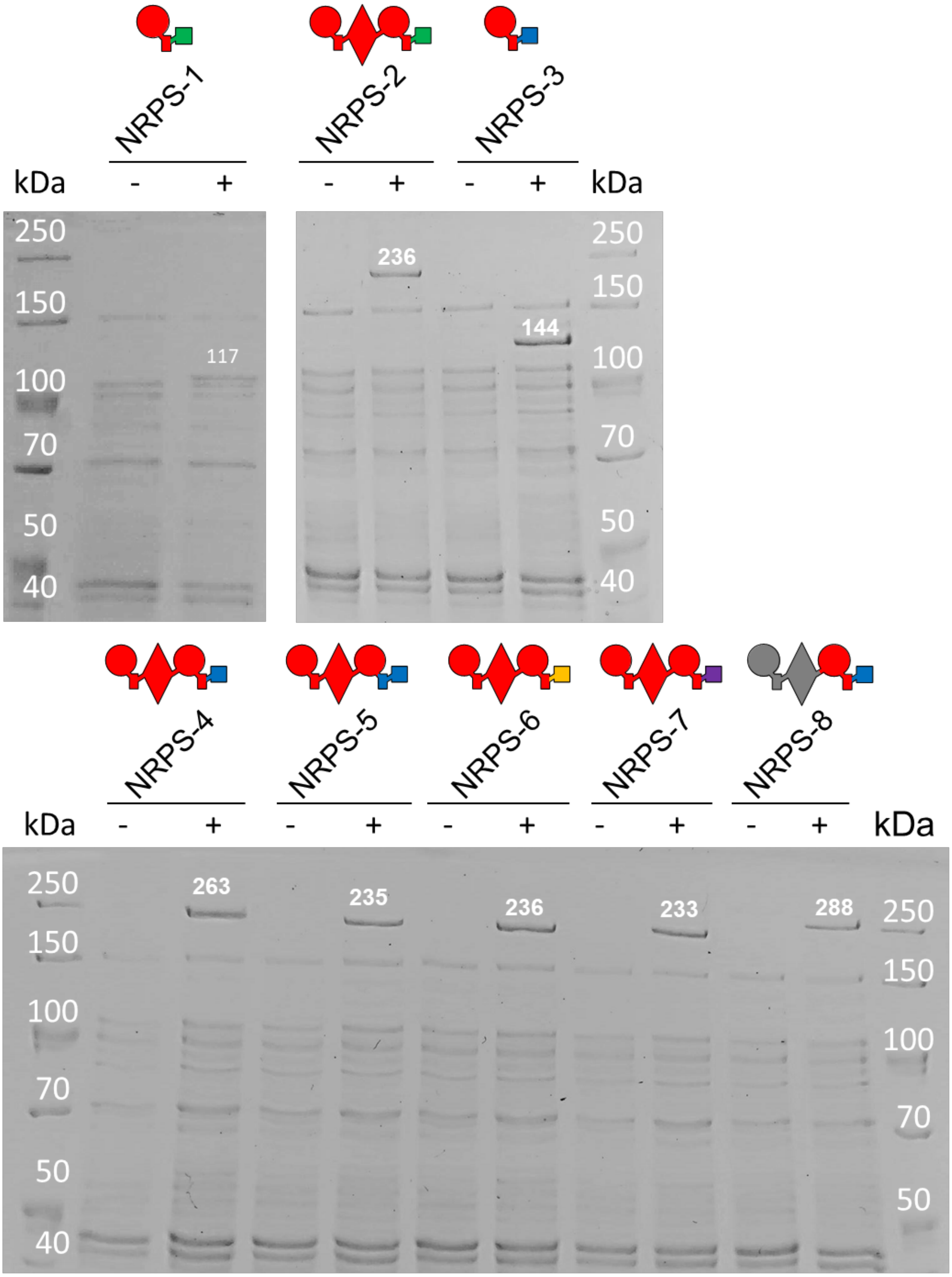

Supplementary Figure 12. SDS-PAGE analysis of engineered proteins. Culture extracts of *E. coli* cells with the respective plasmids after induction with (+) or without arabinose induction (-). The calculated molecular weights of the proteins and the size of the marker proteins are indicated. See Fig. 1 and 2 for assignment of the domain symbols. The colour identifies NRPSs used as building blocks (Supplementary Fig 9).

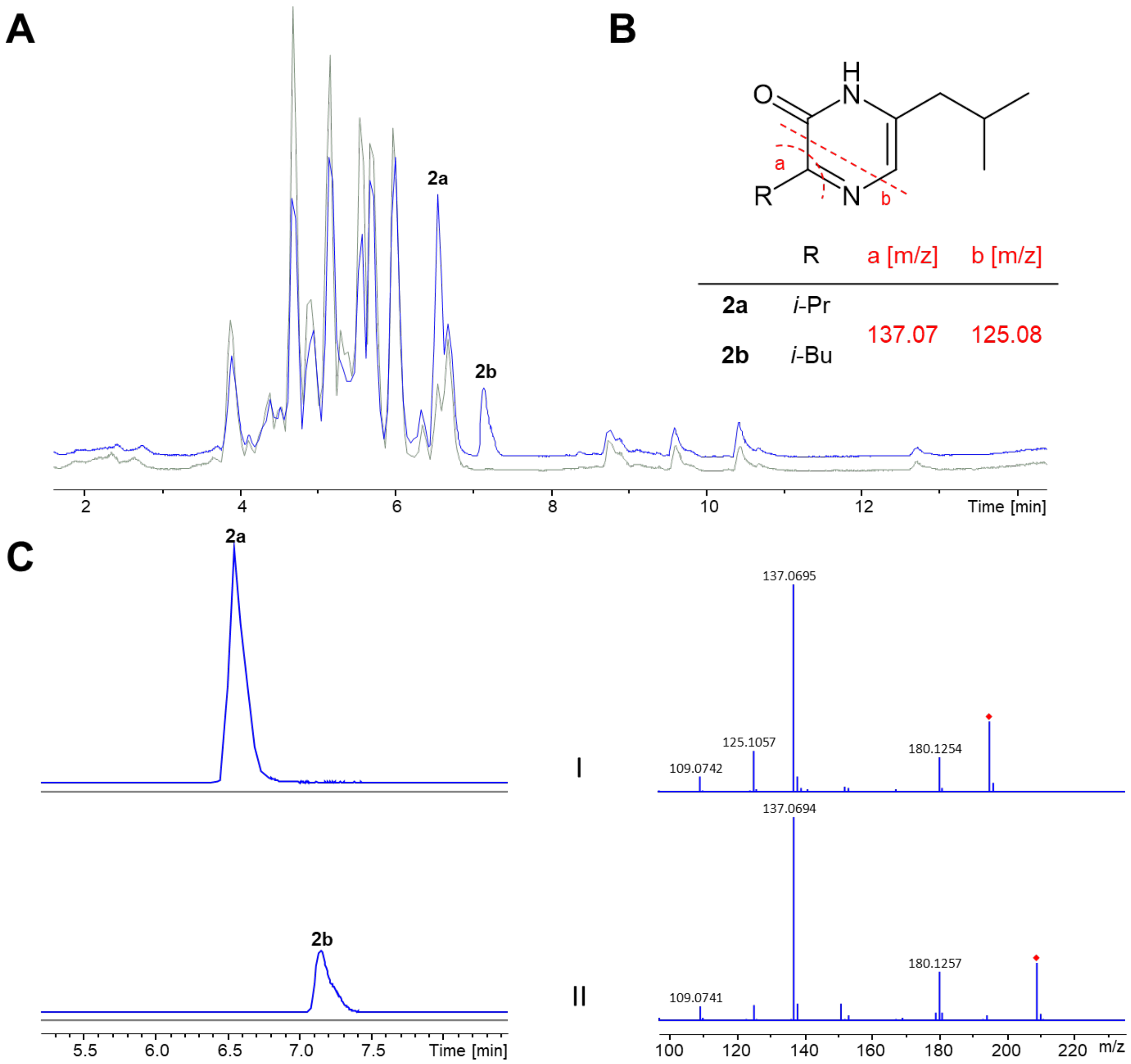

Supplementary Figure 13. HR-HPLC-MS data of **2a** and **2b** produced by NRPS-4 after heterologous expression in *E. coli* DH10B::*mtaA*. **(A)** Stacked BPC of non-induced (grey) and induced (blue) production from NRPS-4. **(B)** Structure of **2a** and **2b** and MS^2 fragments (red). **(C)** Stacked EIC (left) and MS^2 spectra (right) of **2a** (I, rt = 6.6 min, *m/z* [*M*+H$^+$]$^+$ = 195.149; calculated ion formula $C_{11}H_{19}N_2O$; Δppm 1.0) and **2b** (II, rt = 7.2 min, *m/z* [*M*+H$^+$]$^+$ = 209.164; calculated ion formula $C_{12}H_{21}N_2O$; Δppm 1.6).

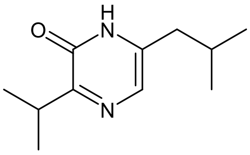

Supplementary Figure 14. Structure of compound **2a.**

Supplementary Table 5. ^{1}H (500 MHz) and ^{13}C (125 MHz) NMR spectroscopic data for compound **2a** in DMSO-d_6 (δ in ppm and J in Hz).

no.	**2a**	
	δ_C	δ_H (mult., J)
1	160.5	
2	156.2	
3	121.4	7.05 (s)
4	138.4	
5	38.8	2.26 (d, 7.3)
6	28.0	1.92 (m)
7	22.4	1.11 (d, 6.9)
8	22.4	1.11 (d, 6.9)
1'	29.7	3.24 (m)
2'	17.6	0.86 (d, 6.6)
3'	17.6	0.86 (d, 6.6)

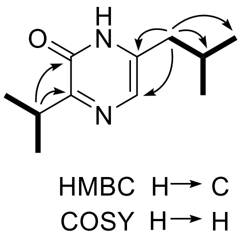

Supplementary Figure 15. Key HMBC and COSY correlations of **2a**.

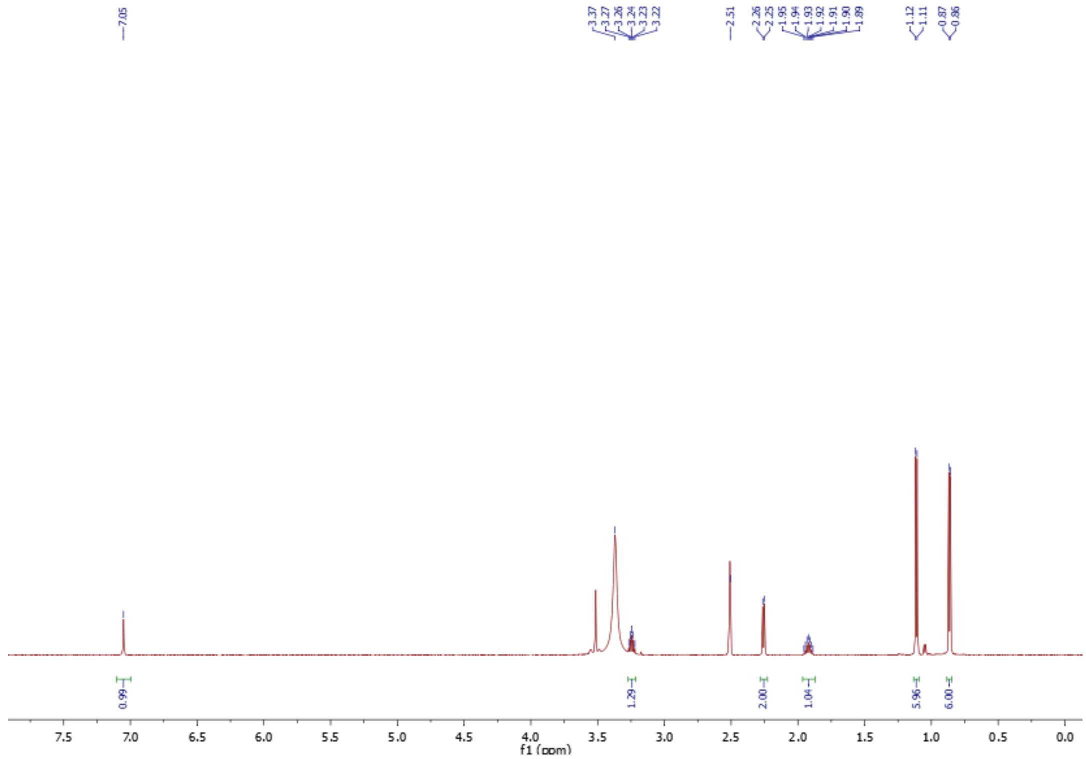

Supplementary Figure 16. ^{1}H NMR spectrum of compound **2a**.

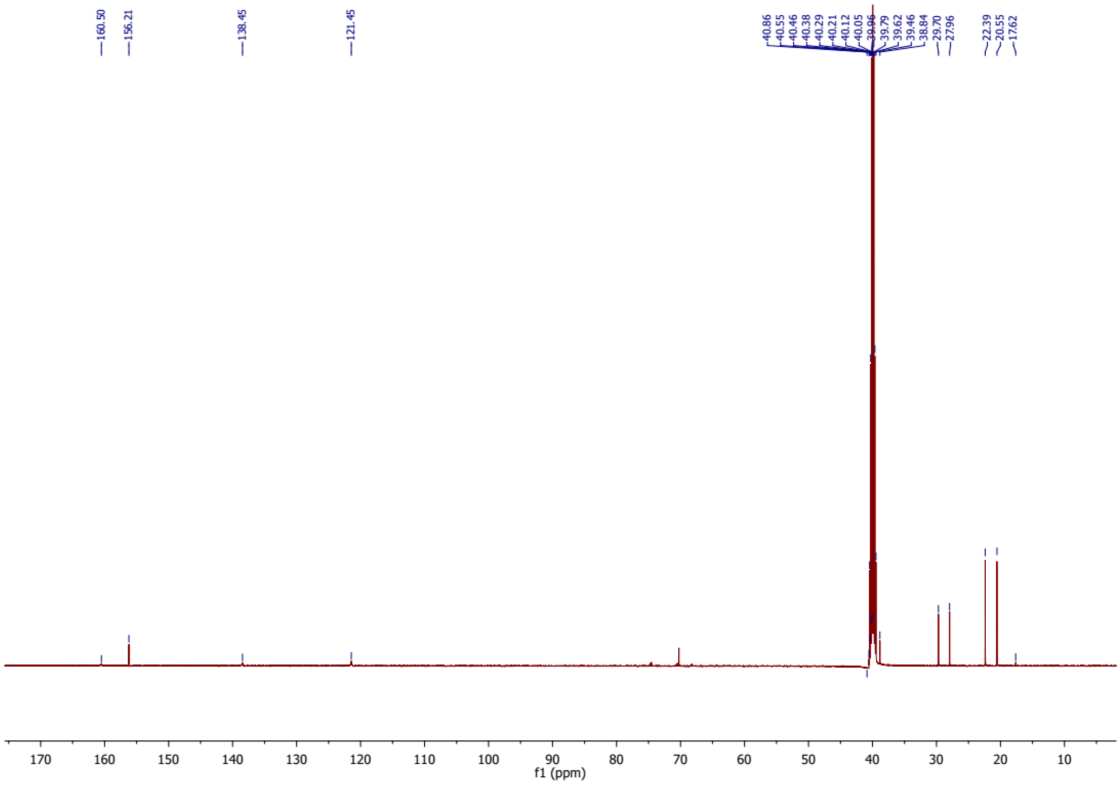

Supplementary Figure 17. ^{13}C NMR spectrum of compound **2a**.

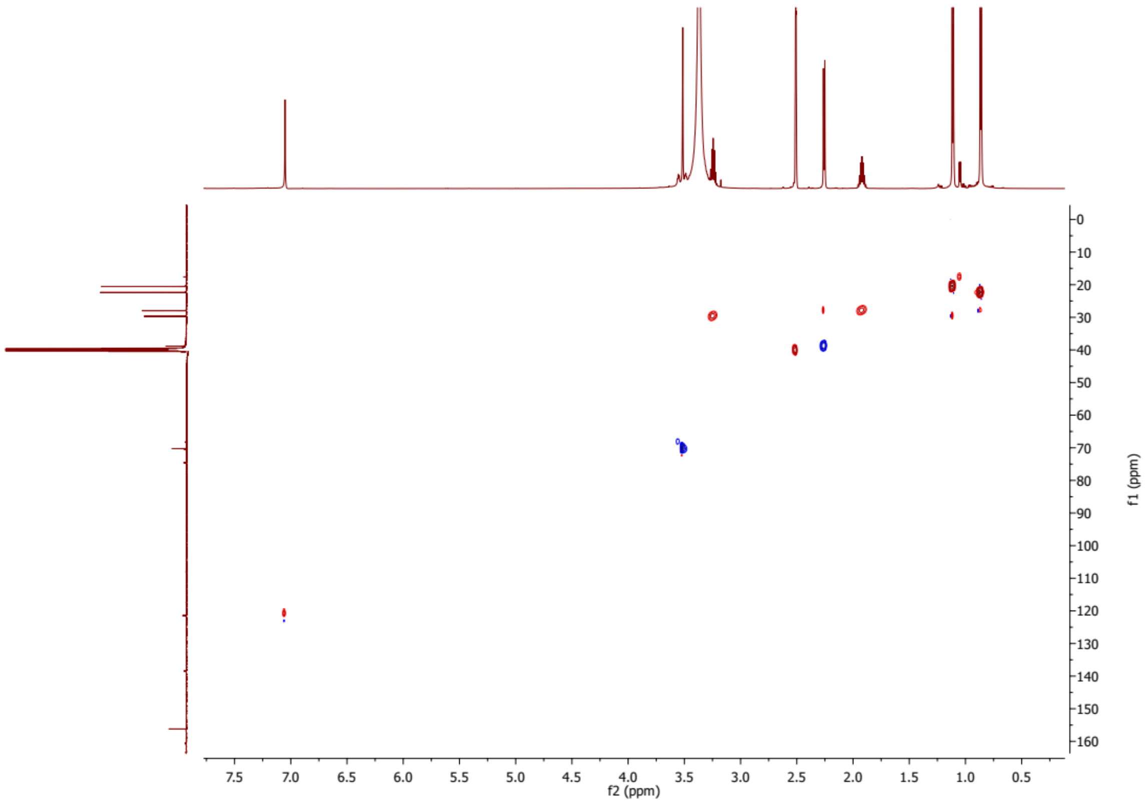

Supplementary Figure 18. HSQC spectrum of compound **2a**.

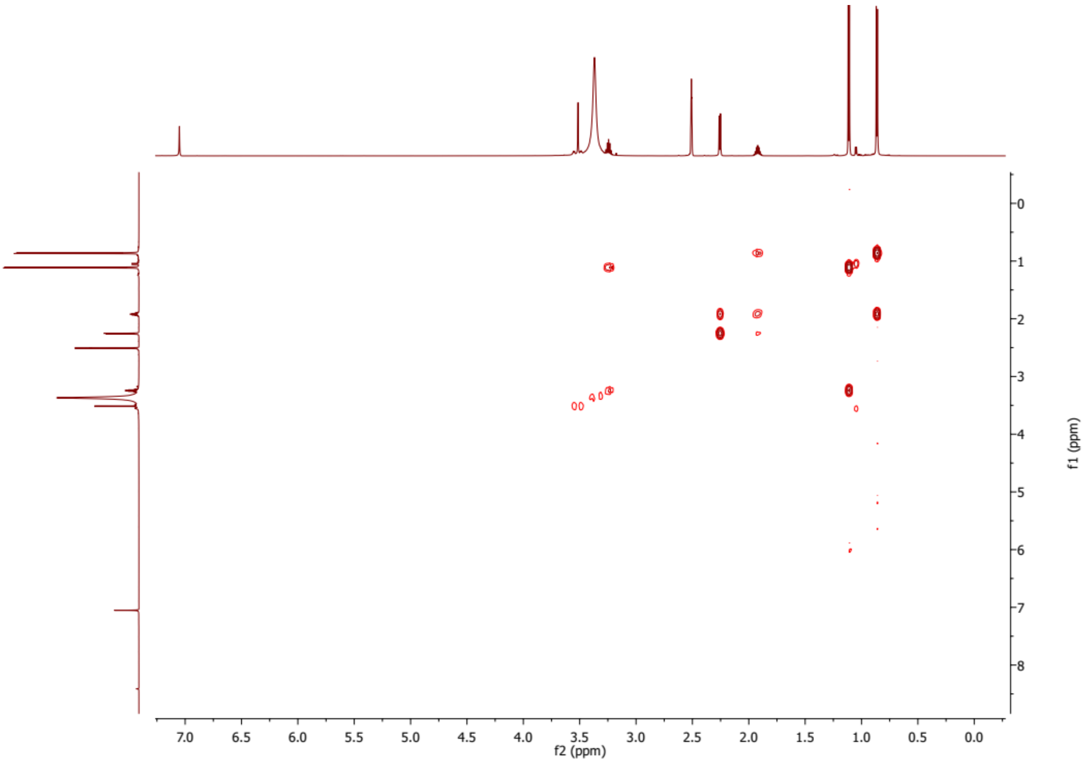

Supplementary Figure 19. COSY spectrum of compound **2a**.

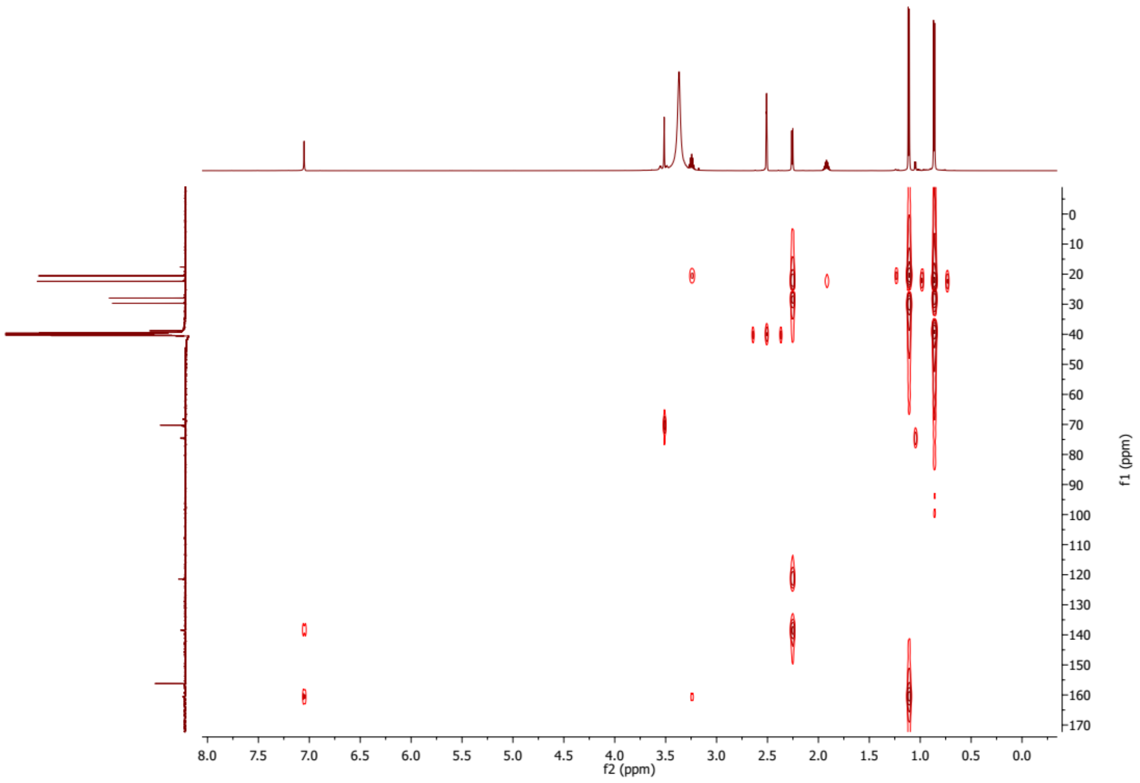

Supplementary Figure 20. HMBC spectrum of compound **2a**.

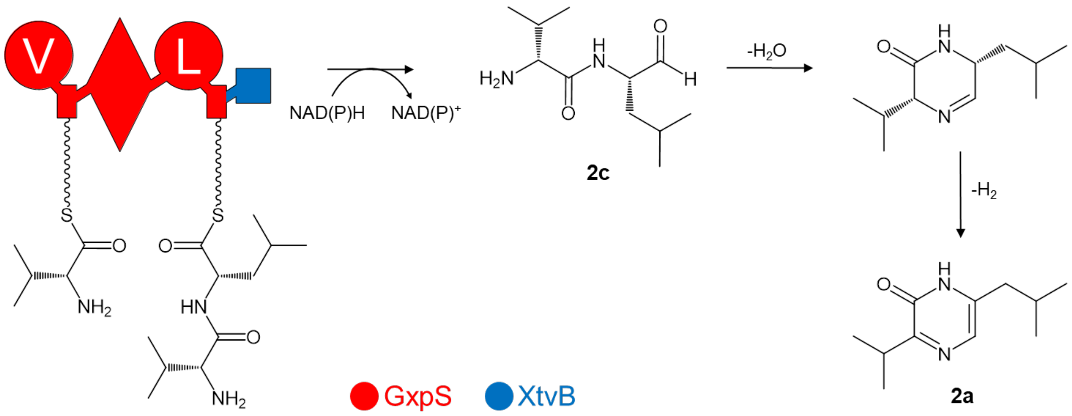

Supplementary Figure 21. Biosynthesis of **2a** by NRPS-4. Standard NRPS biochemistry attaches the nascent D-Val-L-Leu dipeptide on the T2 domain which is released by the R domain via an NAD(P)H-dependent 2-electron reduction of the thioester to produce **2c**. Intramolecular nucleophilic attack of the amino group onto the aldehyde generates a 6-membered Schiff base which oxidizes to yield **2a**. The relaxed substrate specificity of GxpS_A1 can also incorporate Leu beside Val leading to **2b**. See Fig. 1 and 2 for assignment of the domain symbols.The colour code at the bottom identifies NRPSs used as building blocks (Supplementary Fig 9).

A

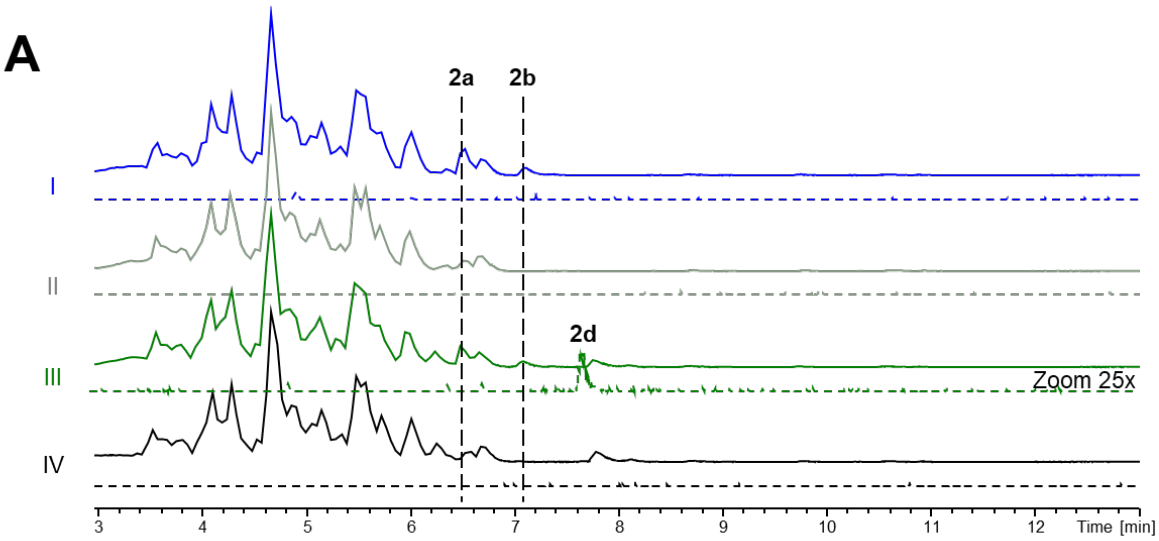

B

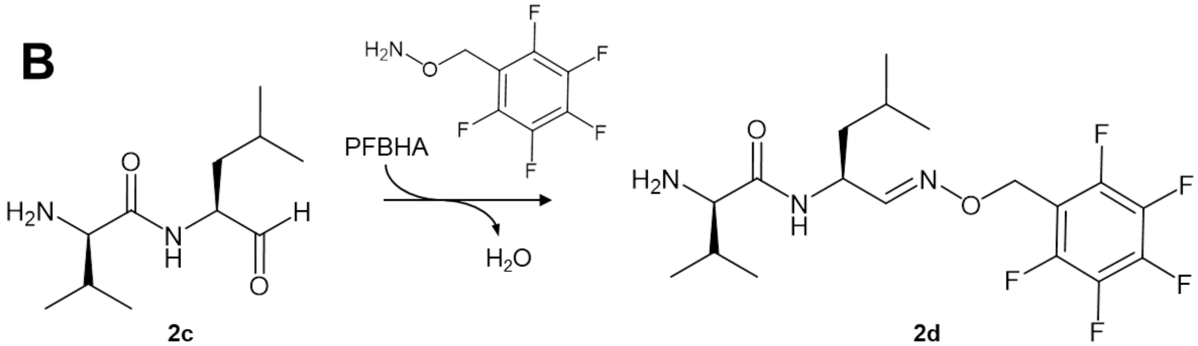

Supplementary Figure 22. (A) HR-HPLC-MS data of **2a** and **2b** as well as derivatization product **2d** of intermediate **2c** produced by NRPS-4 after heterologous production with PFBHA in *E. coli* DH10B::*mtaA*. (I) blue, induced, without PFBHA, (II) grey, non-induced without PFBHA, (III) green, induced with PFBHA and (IV) black, non-induced with PFBHA. The BPC is indicated by continuous lines and the EIC (**2d**; *m/z* $[M+H]^+$ = 410.186; rt = 7.6 min; calculated ion formula $C_{18}H_{25}F_5N_3O_2$; Δppm -1.9) by dashed lines. The y-axes of the EICs are increased 25-fold compared to the BPCs. **(B)** Derivatisation of **2c** with PFBHA resulting in **2d**.

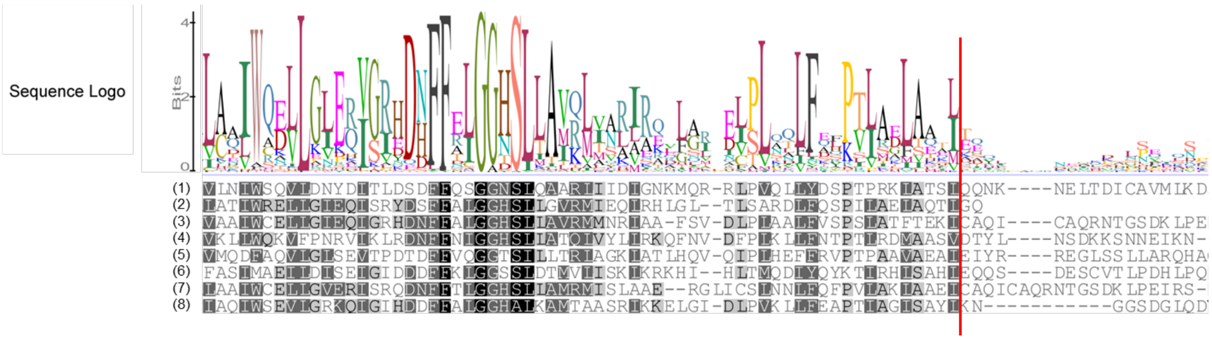

Supplementary Figure 23. Sequence logo and alignment of 86 NRPS T domains. Color code is due to their similarity (black, high similarity; white, low similarity). (1) ATRed_T1 from *X. indica*, (2) GxpS_T1 from *P. luminescens*, (3) GxpS_T2 from *P. luminescens*, (4) XtvB_T2 from *X. eapokensis*, (5) SacC_T3 from *Xenorhabdus* sp. TS4, (6) AusA_T2 from *S. lugdunensis*, (7) GxpS_T5 from pFF1_gxpS_C2$_{int}$[7] and (8) SrfA-C from *B. subtilis*[13]. All sequences are from *Xenorhabdus* and *Photorhabdus* except (6) and (8). The fusion point of T and R domains is indicated by a red line. The data were analyzed with Geneious 6.1.7.

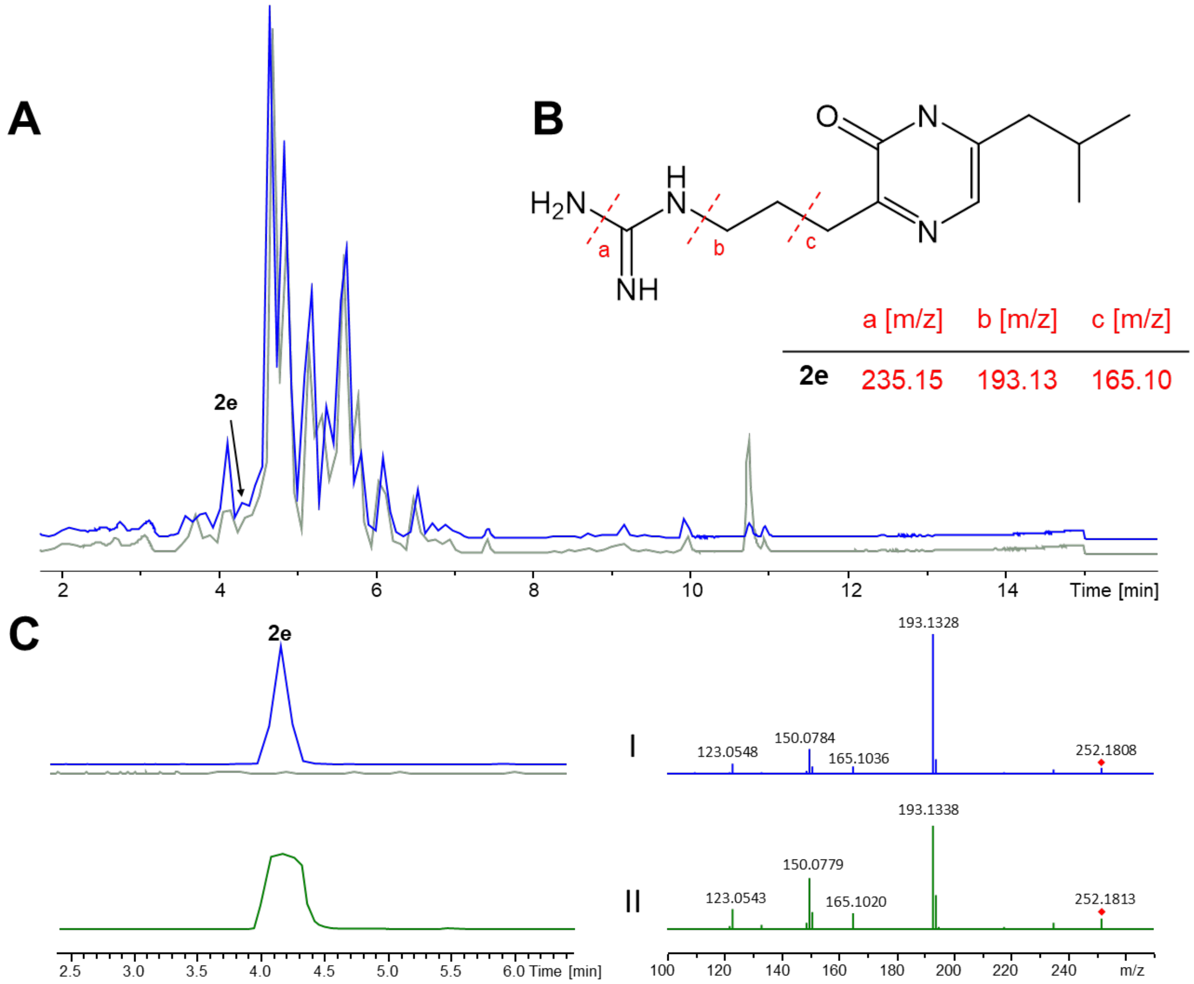

Supplementary Figure 24. HR-HPLC-MS data of compound **2e** produced by NRPS-8 after heterologous expression in *E. coli* DH10B::*mtaA*. **(A)** Stacked BPC of non-induced (grey) and induced (blue) production from NRPS-8. **(B)** Structure of **2e** and MS^2 fragments (red). **(C)** Stacked EIC (left) and MS^2 spectra (right) of **2e** (I, blue, rt = 4.2 min, *m/z* $[M+H^+]^+$ = 252.181; calculated ion formula $C_{12}H_{22}N_5O$; Δppm 1.3) and chemically synthesized **2e** (II, green, rt = 4.2 min, *m/z* $[M+H^+]^+$ = 252.181; calculated ion formula $C_{12}H_{22}N_5O$; Δppm 1.3)

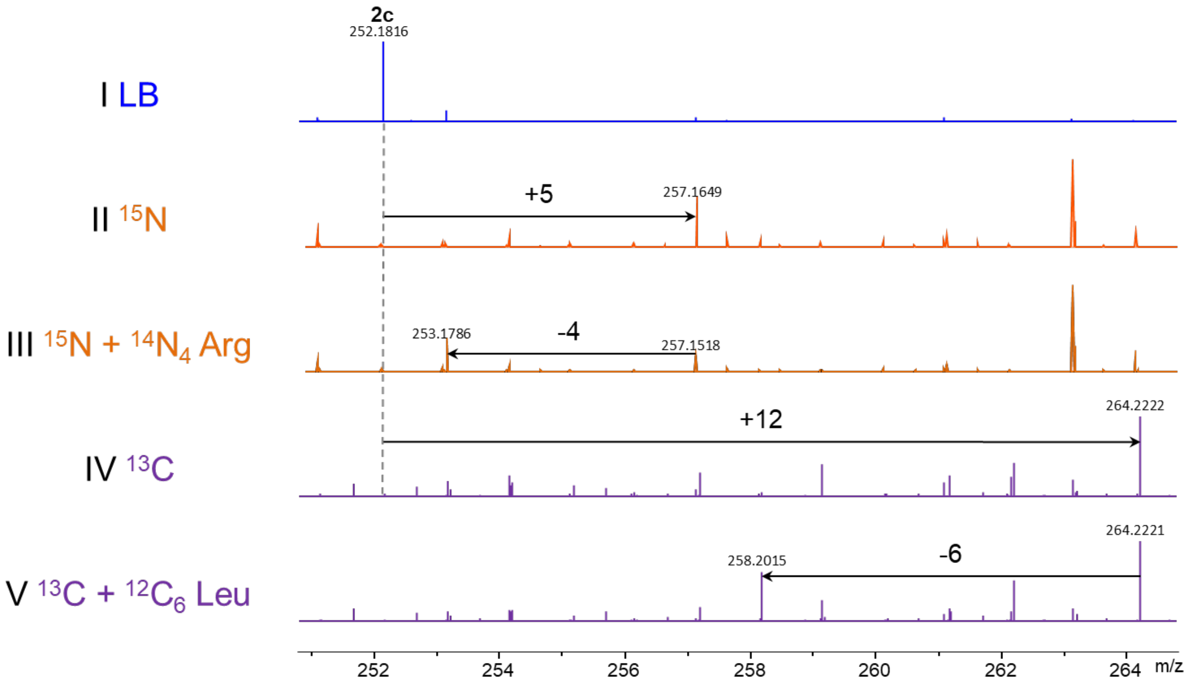

Supplementary Figure 25. Labeling experiments and HR-MS of compounds **2e** produced by NRPS-8 in *E. coli*. MS data of inverse labeling experiments in (I) LB media (blue), (II) ^{15}N media (orange), (III) ^{15}N media supplemented with $^{14}C_4$ Arg (IV) ^{13}C media (purple) and (V) ^{13}C media supplemented with $^{12}C_6$ Leu. The shifts due to incorporation of labelled precursors are indicated by arrows.

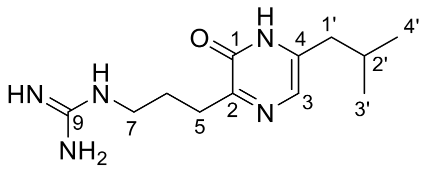

Supplementary Figure 26. Structure of compound **2e.**

Supplementary Table 6. ^{1}H (500 MHz) and ^{13}C (125 MHz) NMR spectroscopic data for compound **2e** in DMSO-d_6 (δ in ppm and J in Hz).

no.	**2e**	
	δ_C, type	δ_H (mult., J)
1	undetected	
2	156.2	
3	121.2	7.05 (s)
4	138.4	
5	29.3	2.65 (t, 7.4)
6	25.6	1.82 (m)
7	40.8	3.16 (m)
8		7.50 (s)
9	157.0	
1'	38.8	2.28 (d, 7.2)
2'	28.1	1.93 (m)
3'	22.4	0.87 (d, 6.2)
4'	22.4	0.87 (d, 6.2)
-NHCO		12.1 (s)

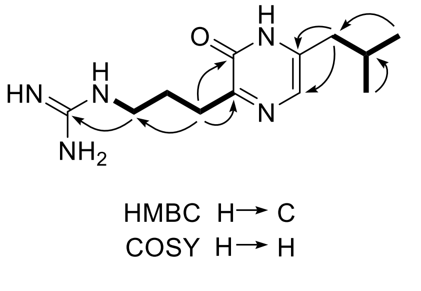

Supplementary Figure 27. Key HMBC and COSY correlations of **2e**.

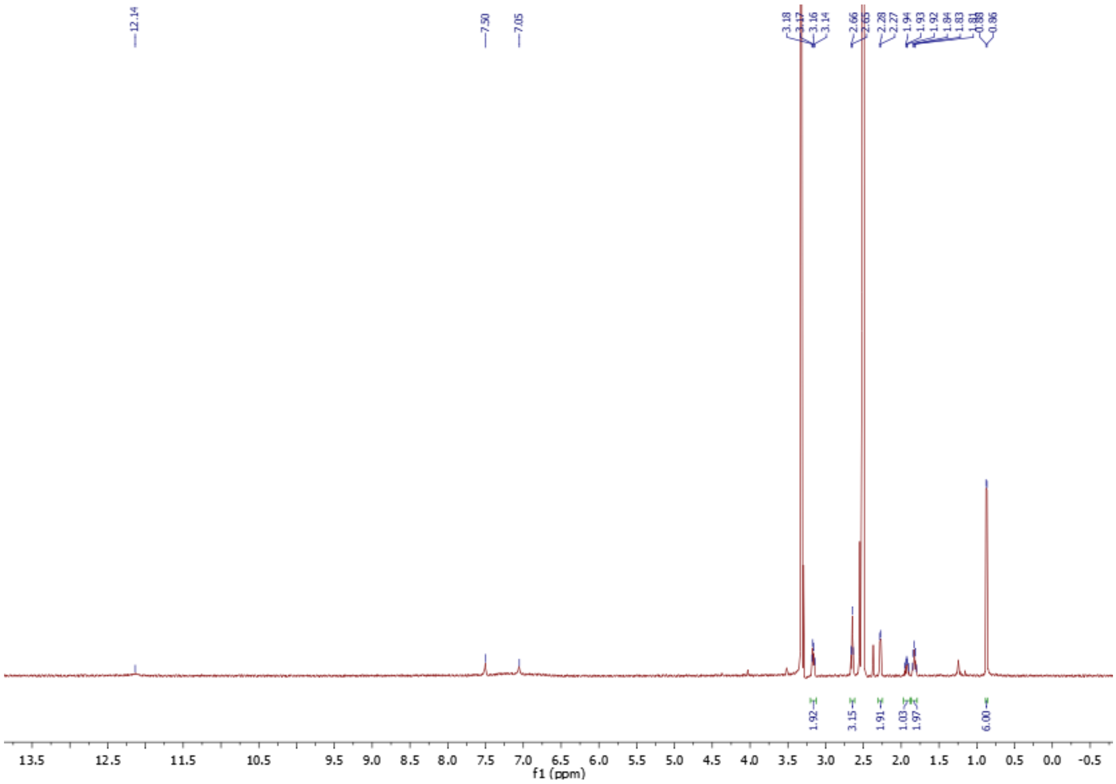

Supplementary Figure 28. ^{1}H NMR spectrum of synthesized **2e.**

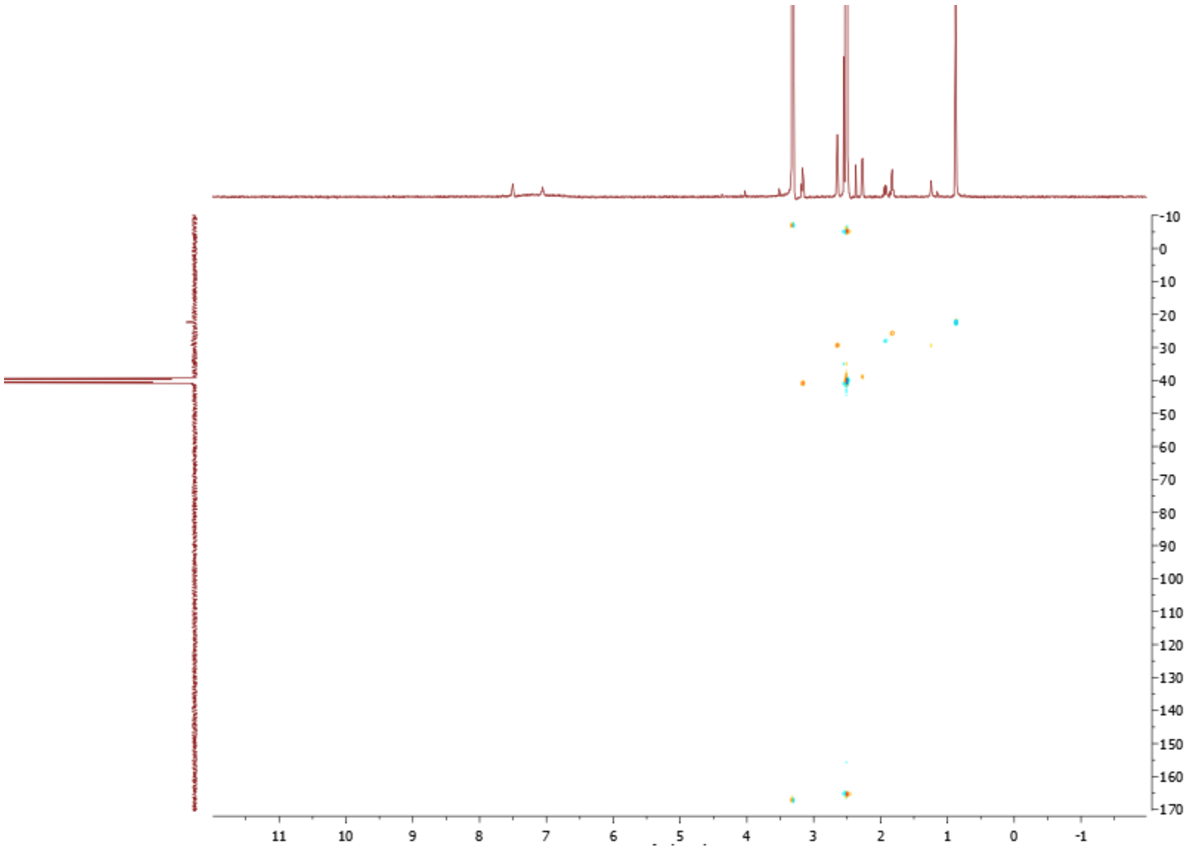

Supplementary Figure 29. HSQC NMR spectrum of synthesized **2e.**

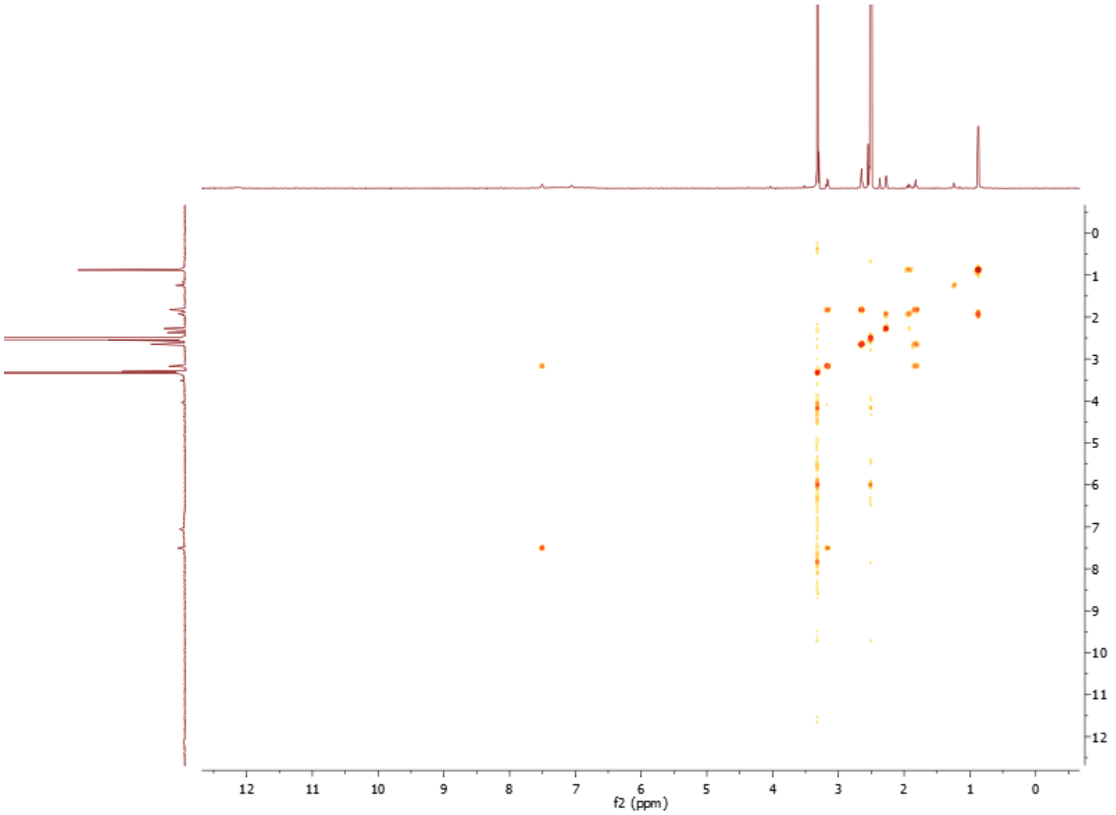

Supplementary Figure 30. COSY NMR spectrum of synthesized **2e.**

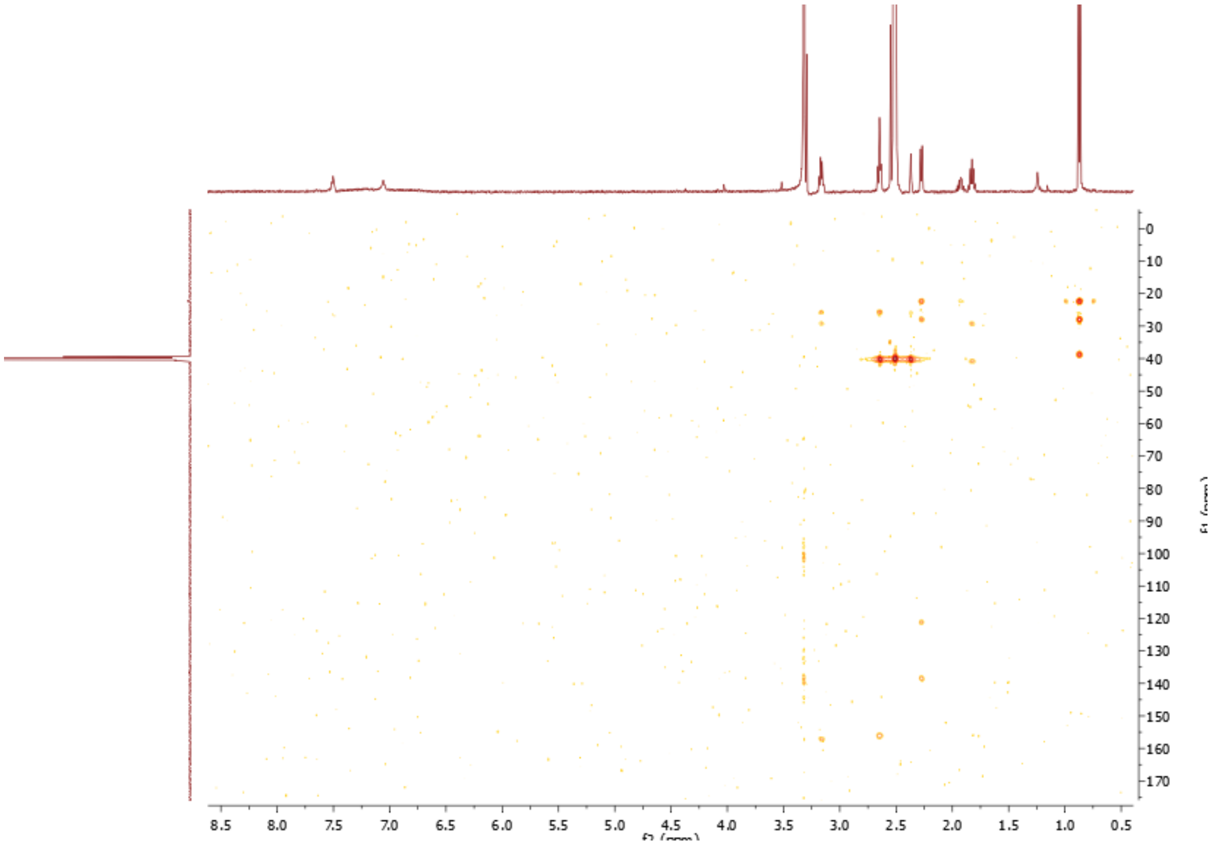

Supplementary Figure 31. HMBC NMR spectrum of synthesized **2e.**

References

[1] D. Hanahan, *J. Mol. Biol.* **1983**, *166*, 557–580.

[2] O. Schimming, F. Fleischhacker, F. I. Nollmann, H. B. Bode, *ChemBioChem* **2014**, *15*, 1290–1294.

[3] S. Thoma, M. Schobert, *FEMS Microbiol. Lett.* **2009**, *294*, 127–132.

[4] K. A. J. Bozhüyük, A. Linck, A. Tietze, J. Kranz, F. Wesche, S. Nowak, F. Fleischhacker, Y.-N. Shi, P. Grün, H. B. Bode, *Nat. Chem.* **2019**, *11*, 653–661.

[5] C. Kegler, F. I. Nollmann, T. Ahrendt, F. Fleischhacker, E. Bode, H. B. Bode, *ChemBioChem* **2014**, *15*, 826–828.

[6] C. Hacker, X. Cai, C. Kegler, L. Zhao, A. K. Weickhmann, J. P. Wurm, H. B. Bode, J. Wöhnert, *Nat. Comm.* **2018**, *9*, 4366.

[7] K. A. J. Bozhüyük, F. Fleischhacker, A. Linck, F. Wesche, A. Tietze, C.-P. Niesert, H. B. Bode, *Nat. Chem.* **2018**, *10*, 275–281.

[8] E. Bode, A. O. Brachmann, C. Kegler, R. Simsek, C. Dauth, Q. Zhou, M. Kaiser, P. Klemmt, H. B. Bode, *ChemBioChem* **2015**, *16*, 1115–1119.

[9] K. Blin, S. Shaw, K. Steinke, R. Villebro, N. Ziemert, S. Y. Lee, M. H. Medema, T. Weber, *Nucleic Acids Res.* **2019**, *47*, W81-W87.

[10] H. B. Bode, D. Reimer, S. W. Fuchs, F. Kirchner, C. Dauth, C. Kegler, W. Lorenzen, A. O. Brachmann, P. Grün, *Chem. Eur. J.* **2012**, *18*, 2342–2348.

[11] H. Wolff, H. B. Bode, *PloS one* **2018**, *13*, e0194297.

[12] S. W. Fuchs, C. C. Sachs, C. Kegler, F. I. Nollmann, M. Karas, H. B. Bode, *Anal. Chem.* **2012**, *84*, 6948–6955.

[13] A. Tanovic, S. A. Samel, L.-O. Essen, M. A. Marahiel, *Science* **2008**, *321*, 659–663.
